# Recent Advances in Flexible Wearable Supercapacitors: Properties, Fabrication, and Applications

**DOI:** 10.1002/advs.202302172

**Published:** 2023-08-03

**Authors:** Zhe Yan, Sheji Luo, Qi Li, Zhong‐Shuai Wu, Shengzhong (Frank) Liu

**Affiliations:** ^1^ School of Materials Science and Engineering Xi'an Shiyou University Xi'an Shaanxi 710065 P. R. China; ^2^ School of Materials Science and Engineering Shaanxi Normal University Xi'an Shaanxi 710062 P. R. China; ^3^ Dalian National Laboratory for Clean Energy iChEM Dalian Institute of Chemical Physics Chinese Academy of Sciences Dalian 116023 China

**Keywords:** energy storage devices, flexible self‐powered devices, flexible supercapacitors, flexible wearable devices

## Abstract

A supercapacitor is a potential electrochemical energy storage device with high‐power density (PD) for driving flexible, smart, electronic devices. In particular, flexible supercapacitors (FSCs) have reliable mechanical and electrochemical properties and have become an important part of wearable, smart, electronic devices. It is noteworthy that the flexible electrode, electrolyte, separator and current collector all play key roles in overall FSCs. In this review, the unique mechanical properties, structural designs and fabrication methods of each flexible component are systematically classified, summarized and discussed based on the recent progress of FSCs. Further, the practical applications of FSCs are delineated, and the opportunities and challenges of FSCs in wearable technologies are proposed. The development of high‐performance FSCs will greatly promote electricity storage toward more practical and widely varying fields. However, with the development of portable equipment, simple FSCs cannot satisfy the needs of integrated and intelligent flexible wearable devices for long durations. It is anticipated that the combining an FSC and a flexible power source such as flexible solar cells is an effective strategy to solve this problem. This review also includes some discussions of flexible self‐powered devices.

## Graphical abstract

In this review, the unique mechanical properties, structural designs, and fabrication methods of each flexible component are systematically classified, summarized, and discussed based on the recent progress of flexible supercapacitors (FSCs). Further, the practical applications of FSCs are delineated, and the opportunities and challenges of FSCs in wearable technologies are proposed.

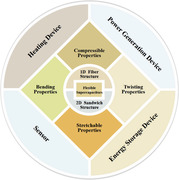

## 1 Introduction

**Figure 1:**
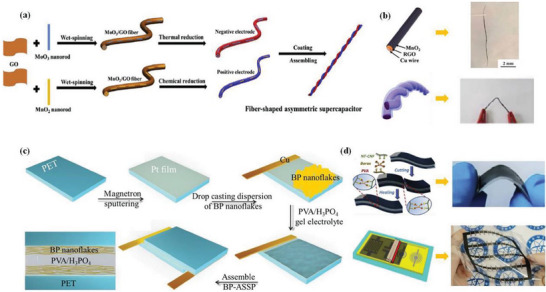
Schematic diagram of the preparation of fibrous and planar FSCs. 1D fiber‐structure supercapacitor a) and photographs of device structures b). Reproduced with permission.^[^ ^5^ ^]^ Copyright 2017, Royal Society of Chemistry. 2D sandwich‐structure supercapacitor c), as well as photographs of device structures d). Reproduced with permission.^[^ ^6^ ^]^ Copyright 2018, Royal Society of Chemistry.

Supercapacitors, also known as electrochemical capacitors, form a promising class of high‐power electrochemical energy storage devices, and their energy density (ED) lies between that of secondary batteries and conventional capacitors.^[^ ^1^ ^]^ According to the particular energy storage mechanism of their electrode materials, supercapacitors can be divided into electric double‐layer capacitors (EDLC) and pseudocapacitors. An EDLC enables the storage and release of electrical energy by rapid adsorption/desorption of ions at the interface between the electrode material and electrolyte.^[^ ^2^ ^]^ Pseudocapacitors perform energy storage and release through fast reversible oxidation/reduction reaction on the electrode surface. However, the conventional supercapacitors encounter an application bottleneck due to their rigid electrodes based on powder materials. Recently, flexible supercapacitive materials have attracted great interest due to their physical, chemical and mechanical properties. Compared with the conventional supercapacitors, FSCs have the greatest advantages of excellent flexibility, low weight, and compression, and can be classified into fibrous FSCs^[^ ^3^ ^]^ and planar FSCs^[^ ^4^ ^]^ on the basis of the various electrode structures(**Figure** **1**).^[^ ^5^, ^6^ ^]^ At present, the challenges of FSCs mainly depend on the introduction of flexible substrates with pseudocapacitance, which has caused the volume and quality of FSCs to increase, and these issues make the devices unsuitable for portable and wearable applications. Because the values depend on all the constituent materials, FSCs have suffered from low specific capacitance and ED. Therefore, the balance between flexibility and specific capacitance is of great significance and needs to be further resolved.

**Figure 2:**
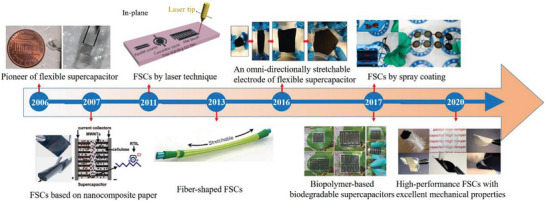
A brief timeline showing the history of the development of various planar FSCs. Inset images: “Pioneer of FSC” Reproduced with permission.^[^ ^11^ ^]^ Copyright 2006, Elsevier. “FSCs based on nanocomposite paper”. Reproduced with permission.^[^ ^12^ ^]^ Copyright 2007, National Academy of Sciences. “FSCs by laser technique”. Reproduced with permission.^[^ ^13^ ^]^ Copyright 2011, Nature Publishing Group. “Fiber‐shaped FSCs”. Reproduced with permission.^[^ ^14^ ^]^ Copyright 2013, Wiley‐VCH. “An omni‐directionally stretchable electrode of FSC”. Reproduced with permission.^[^ ^15^ ^]^ Copyright 2016, American Chemical Society. “Biopolymer‐based biodegradable supercapacitors”. Reproduced with permission.^[^ ^16^ ^]^ Copyright 2017, Royal Society of Chemistry. “FSCs by spray coating”. Reproduced with permission.^[^ ^17^ ^]^ Copyright 2017, American Chemical Society. “High‐performance FSCs with excellent mechanical properties”. Reproduced with permission. ^[^ ^18^ ^]^ Copyright 2020, Royal Society of Chemistry.

Compared with traditional supercapacitors, FSCs not only need to satisfy the basic electrochemical performance requirements, such as wide potential window, high ED with high PD, fast charge/discharge rate, and long cycling stability, but also must have good mechanical deformation ability. Therefore, more stringent requirements are put forward for the electrode, electrolyte, and packaging technology of FSCs. In the past few years, researchers have developed many types of FSCs and are making continuous progress.^[^ ^2^ ^]^ **Figure** **2** shows the history of the development and progress of FSCs. FSCs are typically composed of flexible electrode materials, electrolytes, separators, and encapsulating materials. The main difference from conventional supercapacitors is the flexible electrode material and outer lining in an FSC. Flexible electrode materials are one of the most important components and have a great effect on the performance of FSCs. The typical electrode materials of FSCs are carbon‐based materials, metal oxides, conductive polymers^[^ ^7^ ^]^ and composite electrode materials.^[^ ^8^ ^]^ There are two main methods for preparing flexible electrode materials. One is coating or depositing the active material on a flexible substrate,^[^ ^9^ ^]^ and the other is preparing flexible free‐standing materials, such as flexible films and fibers.^[^ ^10^ ^]^

Among the many supercapacitor electrode materials, carbon‐based materials, such as activated carbon, carbon nanotubes (CNTs), and graphene, have been extensively studied due to their high flexibility, excellent conductivity, and good cycle stability.^[^ ^19^ ^]^ In particular, graphene and CNTs have achieved broader attention because of their high electrical conductivity, high specific surface area and superior performance.^[^ ^20^ ^]^ Although graphene nanosheets have better electronic, physical and chemical properties, they easily stack and agglomerate during the actual preparation process, making the actual capacitance much smaller than the theoretical specific capacitance (550 F g^−1^). For instance, Wang's group^[^ ^21^ ^]^ utilized a microwave‐assisted synthesis of N‐doped‐graphene monoliths (M‐NGM) to create unique nanosheet structures as flexible electrodes that were assembled into solid‐state supercapacitors without current collectors. This method effectively eliminates the re‐stacking of graphene sheets and uncovers the capacitance potential of graphene; thus this method is more effective than loading graphene sheets on a substrate or using graphene powder material. The supercapacitor based on M‐NGM has an areal capacitance of 894 mF cm^−2^ and an ED of 12.2 Wh kg^−1^. It also has excellent cycle stability (∼100% capacitance retention) and mechanical strength (97% after mechanical bending over 500 cycles), because the device has a layered porous structure, good electrical conductivity (15 S cm^−1^) and is rich in nitrogen (6.6 wt.%). Further, Li et al.^[^ ^22^ ^]^ directly prepared graphene fiber fabrics for supercapacitors by a hydrothermal activation method. The areal specific capacitance reached 1060 mF cm^−2^, increased by superposition. Although the carbon‐based materials have shown high flexibility, the specific capacitance has been limited due to the double‐layer adsorption mechanism on the surface of materials. On the contrary, the pseudocapacitive electrode material has higher specific capacitance due to the redox reaction of the bulk phase.^[^ ^23^ ^]^ However, because of the rigid intrinsic properties of metal oxide electrode material, it shows a deficiency when applied in FSCs.^[^ ^24^ ^]^ Therefore, the effective combination of carbon‐based materials and pseudocapacitive material could exhibit a synergistic effect. For example, Zhang et al.^[^ ^25^ ^]^ prepared flexible holey reduced graphene oxide (HRGO) fiber with enhanced electrochemical performance by activating graphene fiber in H_3_PO_4_ solution. After compounding with manganese dioxide, the manganese dioxide/hole graphene fiber supercapacitor electrode material was obtained. An all‐solid‐state FSC was assembled with polyvinyl alcohol (PVA)/H_3_PO_4_ as a gel electrolyte. It had excellent flexibility and an areal specific capacitance of 16.7 mF cm^−2^. Chen et al.^[^ ^26^ ^]^ attached CNTs film to elastic rubber fiber and then deposited a layer of polyaniline (PANI) on the surface of the CNT film by electrochemical deposition to prepare a CNTs/PANI composite flexible electrode. The assembled device exhibited specific capacity of 255 F g^−1^, and was able to maintain 93.8% of the original specific capacitance after 1000 cycles. In short, the selection and preparation of the electrodes play vital roles in the construction of high‐performance FSCs.

In this review, we generalize the characteristics of FSCs and summarize recent advances in the fabrication of FSCs and then provide a comprehensive understanding for new applications of FSCs. Taking into consideration the various properties and fabrication processes, recent progress on FSCs are systematically summarized and discussed based on the various classifications. Finally, we also propose the challenges and outlook for the use of flexible free‐standing supercapacitors as a competitive and innovative strategy for future large‐scale and wide‐ranging energy storage.

## 2 Mechanical Properties, Fabrication, and Applications of FSCs

As is well‐known, the difficulty of FSCs is achieving a balance between flexibility and electrochemical performance. For practical applications, the development direction of FSCs is toward low weight, high ED, long cycle life, good flexibility and compressibility. Here, in order to provide a basis for further study of FSCs, we mainly generalize and discuss the properties, fabrication and applications of FSCs in recent works.

### 2.1 Mechanical Properties of FSCs

FSCs are mainly composed of functionalized electrodes, collectors and electrolyte, which is similar to the common supercapacitors. Considering that their components must be flexible, excellent electrochemical performance of FSCs is realized by 1) applying high‐performance, flexible electrodes, and 2) utilizing a flexible electrolyte and collector. For wearable and portable supercapacitors, good flexibility is as important as electrochemical performance. Based on the different types of components, FSCs can show various properties and abilities, such as compressibility, stretchability, bendability and twisting properties.^[^ ^27^, ^28^, ^29^, ^30^ ^]^ These unique characteristics make FSCs suitable for a wide range of applications. To date, compressible, stretchable, bendable, and twistable supercapacitors have been widely developed, and recent advances of FSCs are summarized in **Table** **1** and **Table** **2**.

**Table 1:** Classification and characteristics of compressible and/or stretchable supercapacitors

Electrodes	Electrolyte	Compression [%]	Stretch [%)]	Capacitance retention	Energy density	Power density	Ref.
N‐doped carbon foams//N‐doped carbon foams	PVA/LiCl	80	–	>97 % (60% compression)	1.35 Wh kg^−1^	2.9 Kw kg^−1^	[31]
N‐doped graphene aerogel//N‐doped graphene aerogel	PVA/KOH	90	–	≈98 % (75% compression)	7.99 Wh kg^−1^	–	[32]
NiCo_2_S_4_/carbon sponge//NiCo_2_S_4_/carbon sponge^[^ ^47^ ^]^	Aqueous KOH	60	–	≈100 % (75% compression)	–	–	[53]
PANI‐single‐walled carbon nanotubes (SWCNTs)‐sponge//PANI‐SWCNTs‐sponge	PVA/H_2_SO_4_	70	–	>97 % [60% compression]	8 Wh kg^−1^	1.5 kW kg^−1^	[34]
CNT‐PPy‐MnO_2_//CNT‐PPy‐MnO_2_	KCl aqueous electrolyte	50	–	>90 % [50% compression]	8.6 Wh kg^−1^	16.5 kW kg^−1^	[35]
Activated charcoal// Activated charcoal	EMIMCl/water gel	90	–	>100% (90% compression)	–	–	[54]
MnO_2_/CNT// MnO_2_/CNT	PVA‐LiCl gel		800	≈90.3% [600% stretch]	213 nWh cm^−1^		[41]
CNTs//CNTs	PAA hydrogel	60	150	≈20% [150% stretch] >100 % [60% compression]			[52]
PPy@CNT// PPy@CNT	PAA/VSNPs hydrogel	80	600	≈350% [600% stretch] >100 % [80% compression]	–	–	[55]
PPy/CNTpaper// PPy/CNTpaper	PAM/ VSNPs hydrogel	50	1000	260% [1000% stretch] 99.4% [50% compression]	–	–	[56]

**Table 2:** Characteristics of bending/twisting supercapacitors

Electrodes	Electrolyte	Bending degree/Twisting state	Capacitance	Cycling performance	Energy density	Power density	Ref.
Activated carbon//NiCoLDH@RGO@copper‐nickel fiber	PVA/KOH	0‐180°	350.9 mF cm^−2^ [1 mA cm^−2^]	90.5% [3000 cycles]	109.6 µWh cm^−2^	749.5 µW cm^−2^	[81]
PANI/RGO fiber hydrogel //PANI/RGO fiber hydrogel	PVA/H_2_SO_4_	Knotted and twisted randomly	112 F g^−1^ [0.08 A g^−1^]	86 % [17000 cycles]	8.80 mWh cm^−3^	30.77 mW cm^−3^	[15]
RGO/MWCNT fiber //MoS_2_‐RGO/MWCNT fiber	PVA/H_2_SO_4_	0‐180° Tightly knotted	5.2 F cm^−3^ [0.16 A cm^−3^]	≈100% [7000 cycles, original‐folded‐original]	>10^−3^ Wh cm^−3^	≈2 W cm^−3^	[82]
MXene nanosheets/GO fiber//MXene nanosheets/GO fiber	PVA/H_2_SO_4_	Tightly knotted	256 F cm^−3^ [0.1 A cm^−2^]	86 % [20000 cycles]	5.1 mWh cm^−3^	700 mW cm^−3^	[84]
MXene/CNT//RuO_2_/CNT fiber yarn	PVA/H_2_SO_4_	0‐180° Tightly knotted	203 F cm^−3^ [2 mA cm^−2^]	90% [10000 cycles] ≈100% [1000 bending cycles of >90°]	61.6 mWh cm^−3^	58 mW cm^−3^	[85]
RGO/carbon fibers//Co_3_O_4_/Ni fiber	PVA/KOH	Twisted randomly	2.1 F cm^−3^ (20 mA cm^−3^)	84% [1000 cycles]	0.62 mWh cm^−3^	1.47 W cm^−3^	[94]
RGO/ stainless steel//Ni‐Co‐S /stainless steel yarn	PVA/LiOH	0‐360° Twisted randomly	127.2 mF cm^−2^ [0.4 mA]	86.2% [3000 cycles].	10.19 mWh cm^−3^	129.1 mWcm^−3^	[95]
MnO_2_/holey RGO// MnO_2_/holey RGO	PVA/H_3_PO_4_	0‐45°	245 F g^−1^ [1 A g^−1^]	80% (1000 cycles)	–	–	[25]
Amino‐ multi‐walled CNT/MnO_2_ film//amino‐ multi‐walled CNT/MnO_2_ film	PVA/LiCl	0‐180°	4.330 F cm^−2^ (5 mV s^−1^]	>90% [2000 bending cycles of 90°] >90% [2000 twisting cycles]	44.57 Wh kg^−1^	337.1 W kg^−1^	[90]
CNTs/MnO_2_/carbon paper// CNTs/MnO_2_/carbon paper	PVA/KOH	0‐180°	123 mF cm^−2^ [1 mA cm^−2^]	91% [200 bending cycles]	4.2 µWh cm^−2^	4 mW cm^−2^	[91]
RGO@MXene//RGO@MXene	PVA/H_2_SO_4_	90° bended twisted	54 mF cm^−2^ [0.1 mA cm^−2^]	≈100% retention [1000 cycles]	–	–	[92]
MoO_3‐x_/TPNF//MnO_2_/TPNF	PVA/LiCl	0–180°	169.6 F g^−1^	90% [5000 cycles]	30.1 Wh kg^−1^	216.5 W kg^−1^	[93]

#### 2.1.1 Compressible Properties

Compression is a common mechanical deformation of FSCs, and a compressible and high‐performance electrode is the key for compressible supercapacitors.^[^ ^31^ ^]^ Design and fabrication of structurally optimized electrode materials are vitally important. In view of both compressibility and high conductivity, flexible carbon materials could have the obvious advantage. For example, Wang et al.^[^ ^32^ ^]^ introduced free‐standing and hydrophilic nitrogen (N)‐doped carbon foams as high‐performance electrodes for compressible supercapacitors. Due to the superior structural flexibility and high porosity of this material, the assembled symmetrical capacitor endured a compressive strain as high as 80%, and no obvious volume reduction was observed at a sustained strain of 55% after compression for 100 cycles. Liu et al.^[^ ^33^ ^]^ utilized a template method to fabricate an arbitrary‐shaped compressible nitrogen‐doped graphene aerogel. The constructed symmetrical supercapacitor not only showed a high ED of 7.99 Wh kg^−1^ at a current density of 1 A g^−1^ but also maintained stable electrochemical performance after 100 compression/release cycles under a compressive strain of 50%. Besides the compressible carbon materials, carbon‐based composite materials containing pseudocapacitive materials (e.g., metal oxides, PANI, transition‐metal dichalcogenides) have also been widely explored, for instance, NiCo_2_S_4_/carbon sponge, PANI‐SWCNTs‐sponge,^[^ ^34^ ^]^ and CNT‐PPy‐MnO_2_,^[^ ^35^ ^]^ all of which displayed prominent electrothermal performance and compressibility by incorporating the compressible carbon materials. In addition, aqueous electrolytes are unfavorable for FSCs under high compression. Because of their high conductivity, semi‐solid state and flexibility, ionic conducting gels are regarded as the main building blocks for these functional devices.^[^ ^36^, ^37^ ^]^

Currently, ionic conducting gels which mainly include of polyvinyl alcohol hydrogels and polymer‐ion liquid gels prepared by simple mixing or the covalent polymerization of vinyl polymer are brittle, so exhibit poor mechanical strength. To solve this issue, Wang et al.^[^ ^38^ ^]^ prepared an EMIMCl (1‐ethyl‐3‐methylimidazolium chloride)/water (20 wt.%) gel through self‐triggered UV polymerization and the continual addition of water. The non‐covalent cross‐linking interaction of this electrolyte can endow the conducting gel with compressive toughness and the ability to self‐recover. The fabricated symmetrical supercapacitors endured 90% compression and even achieved enhanced capacitance under this compression due to the pressure‐induced enhancement of ionic conductivity caused by the change of viscosity. Therefore, the adjustable conducting gels play a vital role in balancing the correlation between the pressure and the electrochemical performance. Gel electrolytes and electrode materials with sufficient flexibility and ability to withstand compressive pressure would allow the fabrication and packaging of the desired FSCs.

#### 2.1.2 Stretchable Properties

Stretchability is also urgently needed for wearable supercapacitors. Some advanced works have focused on flexible yarn‐ or fiber‐based supercapacitors.^[^ ^39^, ^40^ ^]^ In this kind of supercapacitor, the electrodes and electrolytes are two important elements that affect the electrochemical performance and elasticity of the stretchable devices. First, there are many recent reports of research on stretchable electrodes. The structure of stretchable electrodes can be roughly divided into two categories, where one is macro‐scale structure (mandrel coiled, bent, spring fiber, etc.), and the other is micro‐scale structure (e.g., microbuckled or helically wrapped CNT structures).^[^ ^41^, ^42^, ^43^, ^44^, ^45^ ^]^ For instance, Kim et al.^[^ ^41^ ^]^ prepared MnO_2_/CNT‐based ultra‐stretchable supercapacitors by taking advantage of a synergistic structure in which the fiber electrodes are microscopically buckled and macroscopically coiled. These fiber supercapacitors are superelastic (deformation up to 800%). In addition, when the fiber supercapacitors were stretched by 600%, the capacitance maintained ≈90.3% of the initial value. Second, the proper electrolytes are very important for stretchable supercapacitors, even the intrinsically stretchable and compressible supercapacitor. However, the commonly used PVA‐based acidic electrolytes are neither healable nor stretchable, which cause unsatisfactory performance (lower than 100% strains and deteriorated performance at super‐high strain).^[^ ^46^, ^47^, ^48^ ^]^ Therefore, it's essential to develop a multifunctional polyelectrolyte to achieve intrinsic self‐healability, high stretchability and high compression.^[^ ^49^, ^50^ ^]^ Hence, efficient ionic conduction, effective crack‐activated hydrogen bonds crosslinking and reversible crosslinking interactions among polymer chains are required. Hydrogel is a kind of hydrophilic polymer network swollen with a tunable amount of water that is stretchable and compressible. The water confined in hydrogels can dissolve ions, thus making hydrogels good ionic conductors.^[^ ^51^ ^]^ As a result, taking advantage of hydrogel polyelectrolytes in supercapacitors could promote the super‐stretchability, compressibility, and ionic conductivity of supercapacitors. For instance, Yan et al.^[^ ^52^ ^]^ developed a novel electrolyte by using polyacrylic acid dual‐crosslinked by vinyl hybrid silica nanoparticles and hydrogen bonding (PAA‐VSNPs hydrogel). The polyelectrolyte can be stretched to over 3700% strain, and the as‐assembled supercapacitor maintained 350% capacitance under 600% stretching and exceeded 100% capacitance under 80% compression.

Typically, Zhi et al.^[^ ^56^ ^]^ introduced vinyl mixed silica into polyacrylamide hydrogel backbones to form polyacrylamide‐vinyl silica nanoparticles (PAM‐VSNPs) hydrogel electrolyte, which can improve dynamic cross‐linking of the polymer networks. These cross‐linkers can serve as stress buffers to disperse energy as the strain is applied, which provides a solution to the problems of the inherently low stretchability and compressibility of traditional supercapacitors. Thus, the developed supercapacitor exhibited an unprecedented 1000% strain with an enhanced performance of 260% and compression to 50% strain with good capacitance retention of 99.4%.

#### 2.1.3 Bending/Twisting Properties

FSCs can mainly be divided into either the 1D fibered‐shape or the 2D sandwich structure.^[^ ^57^, ^58^, ^59^, ^60^ ^]^ They have shown various unique properties, such as the ability to stretch, bend and twist.^[^ ^61^, ^62^, ^63^, ^64^, ^65^ ^]^ Both bending and twisting properties are exhibited by 1D fibered‐shape electrodes, and 1D CNT‐based and graphene‐based fibers have shown great promise due to their remarkable mechanical properties;^[^ ^66^, ^67^, ^68^ ^]^ however, as they are based on a double‐layer adsorption mechanism, the single‐carbon materials only show moderate energy storage performance in terms of volumetric capacitance, leading to low ED and PD.^[^ ^68^, ^69^, ^70^ ^]^ Attempts were made to increase the volumetric performance of carbon fibers through the addition of pseudocapacitive materials such as conducting polymers, metal oxides, transition‐metal dichalcogenides and MXene.^[^ ^71^, ^72^, ^73^, ^74^, ^75^, ^76^, ^77^ ^]^ However, inclusion of high pseudocapacitive material has a negative effect on the electrochemical or mechanical properties of the fibers, resulting in poor energy‐storage performance. Therefore, there is often a trade‐off between device PD and ED. In the previous works, the coating materials combining carbon and pseudocapacitive materials typically achieved low gravimetric and volumetric performances, which must be higher for wearable applications.^[^ ^78^, ^79^, ^80^ ^]^ In recent years, some effective strategies have been reported to realize the efficient combination of different materials. For example, Lu et al.^[^ ^81^ ^]^ adopted 3D copper foam in conjunction with graphene sheets to connect the active material to a nickel fiber current collector, significantly increasing the specific surface area and decreasing the interfacial resistance. In comparison to the active material directly grown onto the nickel fiber, the assembled asymmetric supercapacitor with this architecture achieved an improvement in areal capacity to 350.9 mF cm^−2^, and retained 95% of its capacitance under bending of 180°. Yu et al^[^ ^15^ ^]^ fabricated a fibrous hydrogel electrode by the macromolecular self‐assembly of PANI and graphene oxide (GO) hydrogels, and a hydrogel‐state symmetrical supercapacitor was assembled based on the obtained electrodes. Such device presented only small performance changes in the normal, knotted, and twisted states, and delivered a considerable volumetric ED of 8.80 mWh cm^−3^ at a PD of 30.77 mW cm^−3^. Chen et al.^[^ ^82^ ^]^ incorporated MoS_2_ and reduced graphene oxide (RGO) nanosheets into neatly aligned multi‐walled CNT (MWCNT) sheet followed by twisting. The fabricated asymmetric supercapacitors (MoS_2_‐RGO/MWCNT fiber//RGO/MWCNT fiber) were able to operate in a wide potential window of 1.4 V and delivered unaltered cyclic voltammetry (CV) performance under bending up to 180°. Both CV and Columbic efficiency were steady with or without bending, even after 7000 cycles, indicating a high robustness of the device. Additionally, spinning technology is also effective for achieving electrodes suitable for high bending/twisting.^[^ ^83^ ^]^ Razal et al.^[^ ^84^ ^]^ utilized liquid crystal‐assisted spinning of Ti_3_C_2_ MXene nanosheets into novel fibers with a high volumetric capacitance of 256 F cm^−3^ and high conductivity of 72.3 S cm^−1^. Unlike other pseudocapacitive materials, for which electrical conductivity is compromised at high loading, they found that MXene could boost the conductivity (up to 2.5 times) even at the highest loading of ≈88 wt.%. These fibers also exhibited exceptional mechanical properties and endured the mechanical stress present during the knitting of textile prototypes. Razal et al.^[^ ^85^ ^]^ has also employed a bi‐scrolling technique to enable the spinning of yarns containing predominantly MXene nanosheets (up to ≈98 wt.%) trapped within CNT yarn scrolls. The assembled asymmetric device (MXene/CNT//RuO_2_/CNT fiber yarn) reached a high capacitance of 203 F cm^−3^ at 2 mA cm^−2^, high capacitance retention of 100% (after 1 000 bending cycles of >90°), a maximum ED of 61.6 mWh cm^−3^, and a PD of 5428 mW cm^−3^.

A 2D‐sandwich‐structured supercapacitor with high areal ED can be applied to a usable area of the human body or to a portable device. Bendability is an important mechanical property to consider when evaluating such a device.^[^ ^86^ ^]^ In particular, a highly bendable, high‐performance electrode plays an important role in these FSCs. One flexible electrode design generally involves electrochemically active electrode materials and a flexible structural support. High true performance of a supercapacitor depends on both the active materials and the inactive components, particularly the current collector or support. In order to provide a feasible amount of energy, High mass loading of active materials is necessary. The common structural supports include polyethylene glycol terephthalate (PET) film, air‐laid papers, carbon‐based materials, metal‐based materials, etc.^[^ ^87^, ^88^, ^89^ ^]^ For example, Cai et al.^[^ ^90^ ^]^ assembled a symmetrical supercapacitor with a PET membrane as the support and free‐standing amino‐multiwalled CNT/MnO_2_ films as electrodes, prepared by electrospinning. This device retained 90% of its capacitance after 2 000 bending cycles at an angle of 90° and exhibited a maximum ED of 44.57 Wh kg^−1^ and a maximum PD of 13330 W kg^−1^ with a working voltage as high as 1.8 V. Xu et al.^[^ ^91^ ^]^ utilized CNTs/MnO_2_ decorated air‐laid papers was used as the electrodes. The electrodes possessed good electrochemical performance and excellent flexibility and tenacity, including after being bent, folded, stretched, in addition to being breathable. The constructed symmetrical supercapacitors could be repeatedly bent from 0°−180°, and their capacitance retention exceeded 91% after 200 bending cycles. Fan et al.^[^ ^92^ ^]^ assembled a sandwich‐structured supercapacitor with carbon cloth as the current collector and MXene@RGO composites as symmetrical electrodes. MXene@RGO composites were obtained by facile plasma exfoliation while incorporating GO into MXene as a binder and expansion agent. The obtained device delivered an areal specific capacity of 54 mF cm^−2^ (0.1 mA cm^−2^) and ≈100% capacitance retention (1000 cycles). More importantly, it could be bent or twisted over 90° with only a slight variation of capacitance. In addition, metal fiber‐based electrodes have recently attracted a lot of attention due to their high conductivity for charge transportation, which enables better electrochemical kinetics and mechanical strength. Yu et al.^[^ ^82^ ^]^ developed a nickel current collector with through‐pore structured, which was prepared by electrodepositing nickel on laser‐drilled stainless steel sheets filled with epoxy resin. Based on this flexible current collector, the electrodes present high conductivity of 2 × 10^6^ S m^−1^ and higher performance than those fabricated utilizing conventional current collectors. At a high active mass loading of 19.4 mg cm^−2^, the supercapacitors exhibited excellent flexibility and high energy densities of 50.4 Wh L^−1^ and 30.1 Wh kg^−1^. In short, based on numerous reports, architecture optimizations of the electrode materials, current collector, electrolyte and the entire device are of great significance for balancing the mechanical properties and electrochemical performance. In particular, the structural design of a free‐standing electrode with high active‐mass loading plays a vital role in ensuring sufficient utilization and that electrolyte ions enter the inner part of the electrode material.

### 2.2 Fabrication of FSCs

Regarding the fabrication of FSCs, the development of flexible electrode materials is the top priority of flexible capacitor design. In this section, recent methods for the preparation of high‐performance, flexible electrode materials, including hydrothermal synthesis, vacuum filtration, deposition, printing, spraying, and spinning, are summarized.

#### 2.2.1 Hydrothermal Synthesis for Flexible Electrodes

**Figure 3:**
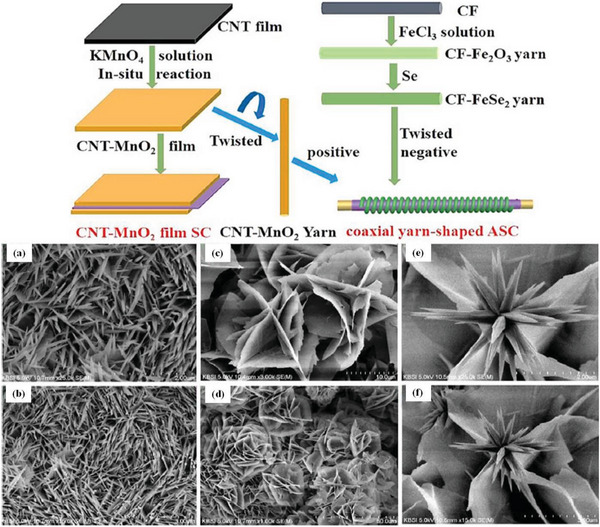
Schematic diagram of a CNT‐MnO_2_ thin‐film, reconfigurable supercapacitor and coaxial yarn asymmetric supercapacitor. Copyright 2019, Elsevier. And the SEM images of prepared NiCo_2_O_4_ with different reagents urea a,b), HMTA c,d), urea‐HMTA e,f), respectively. Reproduced with permission.^[^ ^98^ ^]^ Copyright 2006, Elsevier.

Hydrothermal reaction is a simple preparation method that can control the expected chemical products by regulating temperature and time.^[^ ^96^, ^97^ ^]^ As a typical example, Wang's group^[^ ^16^ ^]^ reported the synthesis of large‐area MnO_2_ nanosheets on 10 µm thin CNT films by hydrothermal reaction. When used as FSC electrodes, the rugged and flexible CNT‐MnO_2_ films exhibited excellent Faradaic pseudocapacitance and could be reconfigured from planar supercapacitors to stretchable thread‐like supercapacitors. A narrow strip of the CNT‐MnO_2_ electrode was also used to construct a new coaxial asymmetric supercapacitor by rolling and twisting the negative electrodes manufactured by synthesizing FeSe_2_ nanotubes on carbon fibers (**Figure** **3** top). The architecture had a high ED of 27.14 Wh kg^−1^ and PD of 571.3 W kg^−1^. It also exhibited excellent flexibility along with long lifetime including 8 000 constant‐current charge‐discharge cycles.

Further, Naresh et al.^[^ ^98^ ^]^ prepared nanostructure NiCo_2_O_4_ electrodes by hydrothermal reaction with different reagents (Figure 3). The hydrothermal reaction can be carried out in one step at high temperature with a minimum of energy, and the morphology and crystallinity of the nanomaterial can be precisely controlled. The NiCo_2_O_4_ sea‐urchin nanostructure prepared using hexamethylenetetramine and urea as reagents displayed excellent electrochemical properties, which has a high capacitance of 1839.3 F g^−1^ at 0.9 A g^−1^ and a PD as high as 40.1 Wh kg^−1^, along with capacitance retention of 87.1% after 2500 cycles.

#### 2.2.2 Vacuum Filtration for Paper‐Type Electrodes

**Figure 4:**
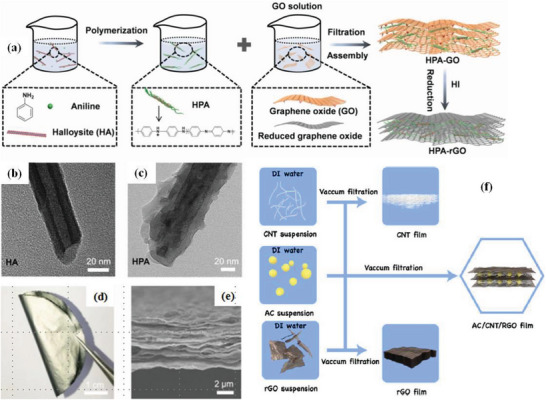
Diagram of the manufacturing process of bio‐inspired nanocomposite films (HPA‐RGO). a) The halloysite‐polyaniline‐graphene oxide nanocomposite films were achieved through polymerization of halloysite(HA) and aniline, mixing of HPA nanocomposites and GO nanosheets, and filtration and reduction, sequentially. TEM images of HA b) and HPA nanocomposites c). A digital photograph d) and a cross‐sectional SEM image e) of HPA‐RGO‐II nanocomposite film. Reproduced with permission.^[^ ^100^ ^]^ Copyright 2019, Elsevier. f) A schematic diagram of the preparation process of the AC/CNT/RGO self‐supporting film, RGO film and CNT film. Reproduced with permission.^[^^101]^ Copyright 2018, Elsevier.

Vacuum filtration is also a simple preparation method, which has great advantages in the preparation of high‐conductivity electrode films. In this method, the active substance is usually mixed into suspension, and the suspension is filtered into filter cake remaining on a filter membrane fitted in a Brinell funnel attached to a vacuum pump.^[^ ^99^ ^]^ The flexibility and specific capacitance of the product are controlled by adjusting the thickness of the filter cake. For instance, Wu et al.^[^ ^100^ ^]^ were inspired by the excellent fracture toughness of nacre arising from its alternating inorganic, organic layered structure and rich interfacial interaction (**Figure** **4**a–e). Their nacre‐inspired halloysite‐polyaniline‐graphene oxide (HPA‐RGO) nanocomposite film prepared by vacuum filtration exhibited excellent high electrical conductivity (397.0 S cm^−1^), tensile strength (351.9 MPa) and long cycle life (≈85% of capacitance retention after 10 000 cycles). In addition, Li et al.^[^ ^101^ ^]^ used a 3D structural composite prepared by ultrasonic filtration of micron‐sized activated carbon (AC), one‐dimensional CNT and 2D RGO mixed dispersion as a supercapacitor electrode (Figure 4f). 1D CNT and 2D RGO can be woven into three‐dimensional porous and flexible frames because of its inherent Vander‐Waals force. The interwoven structure can further anchor the AC particles and form a flexible and self‐supporting membrane electrode. The electron conductivity of the alternating current is significantly improved by the surrounding CNTs, and the presence of the AC particles effectively suppresses re‐stacking of the RGO sheets and agglomeration of the CNTs. The 3D composite electrode exhibited excellent electrochemical performance compared to the pure RGO film and the pure CNT film.

#### 2.2.3 Chemistry/Physical Deposition for Flexible Electrodes

The chemical/physical deposition method generally combines electrochemically active materials with a flexible base through chemical/physical methods.^[^ ^102^, ^103^, ^104^ ^]^ The main deposition processes are divided into chemical vapor deposition (CVD), physical vapor deposition (PVD) and electrochemical deposition.^[^ ^105^ ^]^ For example, Zang et al.^[^ ^106^ ^]^ used CVD technology to prepare graphene mesh film and transferred it to different flexible bases. PVA‐H_3_PO_4_ was used as a gel electrolyte to construct solid supercapacitors to realize the flexibility of the device. Supercapacitors based on polishing cloths exhibited excellent electrochemical performance, providing specific capacitances of up to 8 mF cm^−1^ (267 F g^−1^). The electrode thickness was only 1–7 nm, and the device thickness was less than 1 mm, so the resistance of the device was very low. The device also demonstrated excellent rate capability and capacitance retention of 100% after 1000 charge and discharge cycles. Most importantly, these thin film supercapacitors have excellent flexibility and can be converted into various shapes.

As another example, Chen et al.^[^ ^107^ ^]^ used electrospinning to prepare polyacrylonitrile (PAN) nanofiber grids. After carbonization, polypyrrole (PPy) was electrochemically deposited, and then the graphite oxide coating was reduced to obtain double‐shell electrodes for high‐performance, solid‐state supercapacitors including carbon nanofibers (CNFs)@ PPy@ RGO. Due to the synergistic effect of the three materials and the in‐situ electrochemical deposition of PPy on the surface of CNFs, the electrochemical performance of the composite electrode was significantly improved. The newly designed composite electrode also offered significant superiority over the CNFs@PPy electrode by a ten‐percent increase of the highest specific capacitance from 302.7 F g^−1^ to 336.2 F g^−1^ at 2 mV s^−1^.

#### 2.2.4 Printing and Spraying Microelectrodes

Printing and spraying can be used for preparing materials with low preparation cost, excellent flexibility and modularity. The obtained materials can be designed in shape, mass‐produced and possess other excellent features. Such scalable method generally involves the uniform dispersion of the active material into a suspension ink, which is then applied to a flexible substrate.^[^ ^108^ ^]^ A film can be formed by dipping a flexible substrate into a casting solution followed by spin coating, and the prepared material has a uniform composition and excellent adhesion between phases.^[^ ^109^, ^110^ ^]^ Li et al. developed a simple all‐inkjet printing technique for scalable manufacture of graphene‐based micro‐supercapacitors (MSCs) on a variety of substrates. Specifically, a high‐concentration electrochemically exfoliated graphene (EEG) ink was disposed by a solvent exchange technique to efficiently print thick graphene film (with thickness up to 0.7 µm), which was used as electrodes and current collectors. Moreover, poly (4‐styrenesulfonic acid)‐based polyelectrolyte ink was printed as solid electrolytes that remained functional for long periods of time without packaging. The resultant fully printed MSCs showed an areal specific capacitance of 0.7 mF cm^−2^, which is significantly higher than the highest value for graphene‐based MSCs reported previously (0.1 mF cm^−2^). Furthermore, the entire printing process is very simple and can be arbitrarily connected, making it easy to integrate MSCs in any size.

**Figure 5:**
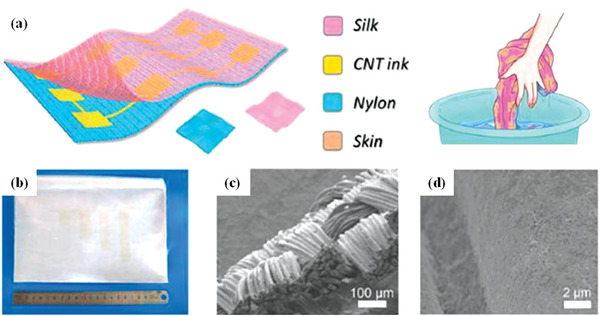
Structural design and sensing mechanism of the washable electronic textile. a) Schematic diagram of the WET with an array of CNT electrodes formed by screen printing. b) Digital photograph of the large‐scale washable electronic textile. c,d) SEM images of the surface of the textile with CNT ink on it at different magnifications, respectively. Reproduced with permission.^[^ ^111^ ^]^ Copyright 2018, American Chemical Society.

As another example, Cao et al.^[^ ^111^ ^]^ used conductive CNTs and screen‐printing technology to create an electronic textile that solved the problems of breathability, lability and mass production on most common fabrics (**Figure** **5**). The use of a flexible textile substrate with CNTs led to excellent performance under extreme mechanical deformation. The washable electronic textile (WET) electrode could be used in an intelligent human‐machine interface, and when integrated with a nanogenerator, the device performed as an electric gesture‐sensing textile, demonstrating its potential application as a self‐powered smart human‐machine interface. Such electronic textiles have high electrical conductivity (0.2 kΩ sq^−1^), high air permeability (88.2 mm s^−1^) and can be manufactured on a large scale on ordinary fabrics.

#### 2.2.5 Spinning for Fiber‐Type Electrodes

**Figure 6:**
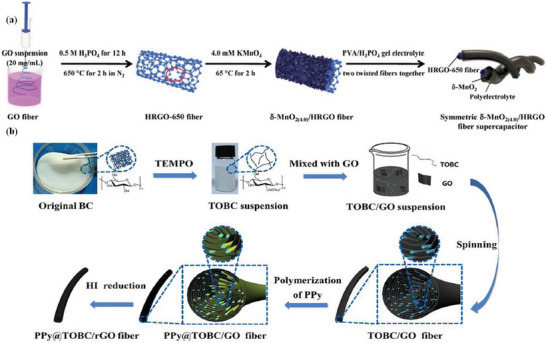
a) Schematic diagram of a wire‐shaped supercapacitor fabricated from two twined d‐MnO_2(4.0)_/HRGO fibers with polyelectrolyte. Copyright 2016, Royal Society of Chemistry. b) Schematic illustration of the fabrication process and structure of PPy@TOBC/RGO macro‐fibers. Reproduced with permission.^[^ ^116^ ^]^ Copyright 2019, Elsevier.

The common spinning methods are mainly wet spinning and electrospinning. Wet spinning mainly consists of disposing the active material into a dispersion liquid and injecting it into a coagulating liquid using a syringe pump, followed by drying to obtain a fiber. The electrospinning method is a special fiber manufacturing process. The polymer solution or melt is spray‐spun in a strong electric field.^[^ ^112^ ^]^ This method has the advantages of simplicity, compatibility with a wide selection of materials and continuous fiber preparation.^[^ ^113^, ^114^ ^]^ Xu et al.^[^ ^115^ ^]^ first reported the production of graphene oxide fibers with high tensile strength and good flexibility by wet spinning, and the prepared graphene oxide fiber could be woven and knotted while having good electrical conductivity. This study laid the foundation for the preparation of graphene oxide fibers by wet spinning. Zhang et al.^[^ ^25^ ^]^ prepared a composite fiber electrode by depositing δ‐MnO_2_ on the surface of HRGO fiber, showing good flexibility and excellent electrochemical performance (**Figure** **6**a). The loosely wound manganese oxide nanosheets self‐assembled on the surface of the HRGO fiber, causing manganese oxide nanosheets to be in intimate contact with the graphene nanosheets, so the composite electrode exhibited excellent specific capacitance of 245 F g^−1^ at a current density of 1 A g^−1^ (in 1 M Na_2_SO_4_). At the same time, two identical fiber electrodes cured by PVA‐H_3_PO_4_ gel electrolyte were intertwined to assemble an all‐solid‐state fiber FSC, showing excellent flexibility, high areal specific capacitance (16.7 mF cm^−2^ at 0.05 mA cm^−2^) and good cycle stability (≈80% of capacitance retention after 1000 cycles). To boost the performance, Sheng et al.^[^ ^116^ ^]^ developed a simple method to prepare flexible, binder‐free high performance all‐solid‐state FSCs based on polypyrrole@TEMPO(2,2,6,6‐Tetramethylpiperidoxyl)‐oxidized bacterial cellulose/RGO (PPy@TOBC/RGO) macrofibers (Figure 6b). The TOBC/GO hybrid fiber was first injected from the needle into the coagulation bath by wet spinning and then dried. PPy improved the electrochemical performance by in‐situ polymerization on TOBC/GO blended fibers, where RGO was used as an electric double‐layer capacitor active material after reduction in hydroiodic acid (HI), and PPy was used as a pseudosupercapacitive material to further improve the specific capacitance. The three components showed a synergistic effect, so that the mixed electrode material had excellent specific capacitance. At a current density of 0.5 A g^−1^ (0.48 A cm^−3^), the specific capacitance was 391 F g^−1^ (373 F cm^−3^). In addition, the fiber‐based supercapacitors had a high ED of 8.8 mWh cm^−3^ at a PD of 49.2 mW cm^−3^, which is superior to the previously reported graphene fiber supercapacitors. An ideal combination of excellent electrochemical performance and good flexibility was achieved.

### 2.3 Applications

**Figure 7:**
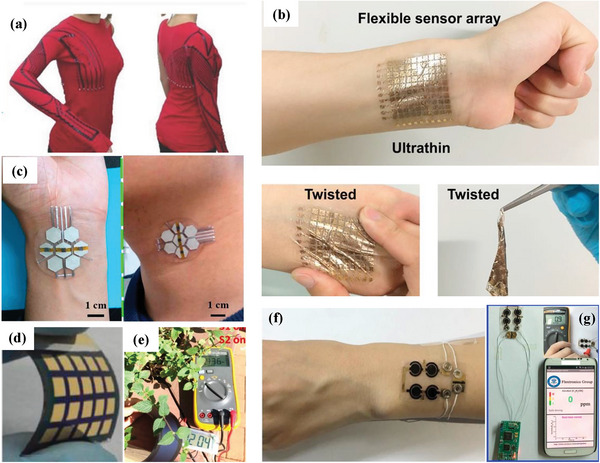
a) CNT strain sensor for human motion detection monitoring in daily activities by fabric pressure sensors. Reproduced with permission.^[^ ^117^ ^]^ Copyright 2014, Wiley‐VCH. b) Optical image of a wearable ultrathin e‐skin sensor array on the wrist and under various mechanical deformations. Reproduced with permission.^[^ ^118^ ^]^ Copyright 2017, Elsevier. c) A multifunctional electronic skin device containing a pressure sensor attached to a wrist and neck. Copyright 2017, Elsevier. d) Optical photo of a flexible perovskite solar cell. Reproduced with permission.^[^ ^120^ ^]^ Copyright 2017, Elsevier. e) Digital optical image of a device assembled with a flexible solar cell and a supercapacitor shows successful energy storage. Reproduced with permission.^[^ ^119^ ^]^ Copyright 2018, American Chemical Society. f) Digital photograph of a wearable MSC array‐gas sensor and PCB on a wrist. g) Real‐time C_2_H_5_OH concentration analysis/display in the case of unknown gas concentration. Reproduced with permission.^[^ ^120^ ^]^ Copyright 2017, Elsevier.

Wearable technology has received widespread public attention and has enormous economic and social impacts that have led to changes in medical procedures and personal lifestyles. Flexible electronic technology represents one of the innovative development directions of wearable technology. Flexible electronic devices have the advantages of ultra‐thinness, good mechanical flexibility and high integration, so they have promise for applications in many fields. To date, researchers have developed flexible devices with a variety of structures and functions, such as flexible sensors (**Figure** **7**a),^[^ ^117^ ^]^ electronic skin (Figure 7b,c),^[^ ^118^, ^120^, ^147^ ^]^ flexible solar cells (Figure 7d,e),^[^ ^81^, ^119^ ^]^ flexible detectors (Figure 7f,g),^[^ ^120^ ^]^ etc. In the following section, we will present some advances in the emerging applications of FSCs for wearable electronics that have occurred in recent years.

#### 2.3.1 FSCs in Energy Storage Devices

**Figure 8:**
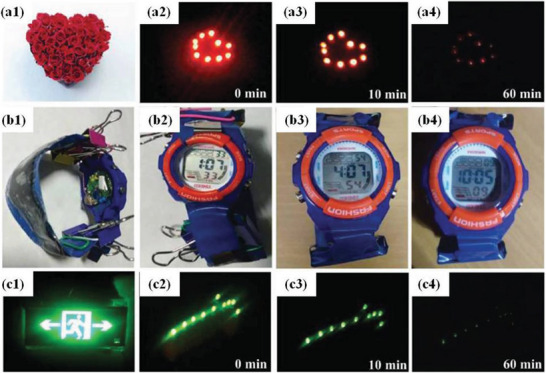
Large‐sized supercapacitor (LSSC) of size 2 × 10 cm^2^ based on 3‐CC (CC electrochemically treated for 3 min). Heart‐shaped image composed of a1) red roses and a2–a4) heart shaped logo consisting of 10 red light emitting diodes (LEDs) in parallel and lit by a single LSSC. b1) Electronic watch with the LSSC as the watchband. (b2–b4) Electronic watch powered by the LSSC watchband. c1) Image of a safety sign and c2–c4) safety indication arrow composed of 10 green LEDs in parallel and lit by the LSSC connected in series to two small devices connected in parallel. Reproduced with permission.^[^ ^123^ ^]^ Copyright 2017, Wiley‐VCH.

Since the yarn or fiber supercapacitors of one‐dimensional structural energy storage units can be easily incorporated into flexible wearable devices in any shape, one‐dimensional supercapacitors have been intensively studied recently.^[^ ^121^, ^122^ ^]^ For example, Qin et al.^[^ ^123^ ^]^ produced a carbon cloth rich in N/O by using a wet‐spinning method. After activation by potassium hydroxide, the flexibility of the carbon‐fiber cloth did not change substantially, the specific surface area was significantly increased and the pore structure was distributed uniformly (**Figure** **8**).

**Figure 9:**
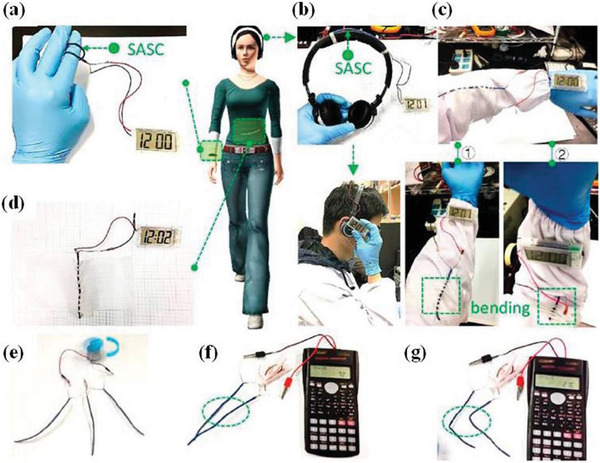
Practical application of the supercapacitor packaged with various electronic devices. a) The curved solid asymmetrical supercapacitor (SASC) powered a digital time display. b) The device was integrated with headphones. c,d) the device was weaved into our lab cloth to power electronics under various bending deformations: ① (straight), ② (bending). e–g) a fan motor and a pocket calculator were powered with the SASCs. Reproduced with permission.^[^ ^81^ ^]^ Copyright 2018, American Chemical Society.

**Figure 10:**
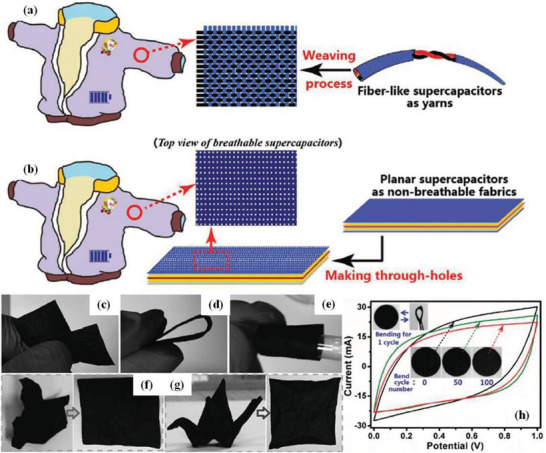
Schematic diagrams of the production of breathable and smart garments through two strategies: a) weaving flexible fiber‐like supercapacitors into a textile, and b) making numerous through‐holes on flexible planar supercapacitors. P4C samples were immersed in 0.1 m KnMO_4_ neutral aqueous solution for 1 min to fabricate P4C/MnO_2_ composites. The obtained P4C/MnO_2_ composites referred to as PCM papers c) kinked, d) bent, e) wound on a pipette dropper, f) crumpled into a ball, and g) folded into a paper crane. Reproduced with permission.^[^ ^91^ ^]^ Copyright 2016, Wiley‐VCH.

The obtained carbon cloth electrode had excellent electrochemical performance. Generally, 1 M Na_2_SO_4_ was used as the electrolyte, and the treated carbon fiber exhibited a stable operating voltage of 2 V, and the specific capacitance as a positive electrode was 2.36 F cm^−2^. The specific capacitance was 1.7 F cm^−2^ as a negative electrode. Compared to other carbon materials, all‐solid‐state carbon‐based symmetric supercapacitors are manufactured to provide ultra‐high ED and excellent flexibility in both acidic and alkaline electrolytes.^[^ ^124^ ^]^ In order to further promote its practical application, the all‐solid‐state FSC was integrated, and a strap‐driven electronic watch lasted up to 9 h, power was supplied to LEDs in a heart‐shaped pattern for ≈1 h, and a safety indicator light was supplied for ≈1 h. This showed that these supercapacitors have great application prospects. As another example, Gao et al.^[^ ^81^ ^]^ converted low‐cost, wasted nickel fiber, cellular copper foam, graphene sheets, and layered double hydroxides into flexible fiber supercapacitors (FFSCs) with high areal capacity and rate performance. Through a series of electrodeposition and dip‐coating operations, 3D graphene skeleton was effectively cloned into nickel fiber. Such FFSCs showed an unprecedented ED of 109.6 µWh cm^−2^ at 749.5 µW cm^−2^, and the device was successfully coupled with flexible strain sensors, solar cells and nanogenerators, proving its potential for use in a variety of commercial and specialty electronic devices. Notably, the solid‐state asymmetrical supercapacitor (SASC) was easily packaged with different devices, woven into a cotton shirt, and even bent to fit the human body. With just 10 s of charging, the curved SASC around the finger successfully powered a digital time display for more than 25 min (**Figure** **9**a). The device was also integrated with a headset to further demonstrate its ability to be wearable and adapt to our body (Figure 9b). In addition, it was built into lab fabrics for use in powering electronic equipment (Figure 9c). By using multiple supercapacitors in series, the operating potential can be increased accordingly to meet the demand of power devices requiring high ED. As shown in Figure 9f,g, a motor fan and pocket calculator could also be powered by the SASC. It is well known that good breathability is often an essential feature of comfortable clothing, yet the breathability of wearable energy storage devices such as smart garments is rarely addressed. As shown, Dong et al.^[^ ^91^ ^]^ used a highly flexible paper electrode to make a supercapacitor that was breathable and wearable (**Figure** **10**a,b). They chose air‐laid paper as a flexible structural support and then deposited CNT and MnO_2_ as electrochemically active materials to prepare paper electrodes. This kind of electrode exhibits good electrochemical properties and enough flexibility to be repeatedly bent, stretched, kinked, pleated into balls, freely cut, and even folded into paper cranes.

In particular, when the paper electrode was subjected to various deformations, the high flexibility of the paper electrode enabled it to maintain relatively stable electrochemical performance. In addition, flexible solid‐state supercapacitors based on paper electrodes and PVA/KOH gel electrolytes showed almost constant capacitance under different bending conditions. After making multiple though holes in the supercapacitors, they became as permeable as normal clothes while retaining 94% of the capacitance. The resulting permeable supercapacitors were still very flexible, and they could be bent repeatedly between 0°−180°, with their retention rate exceeding 91% after 200 bending cycles. This research took a new step for the practical application of wearable and breathable energy storage devices.

#### 2.3.2 FSCs in Power Generation and Heating Devices

**Figure 11:**
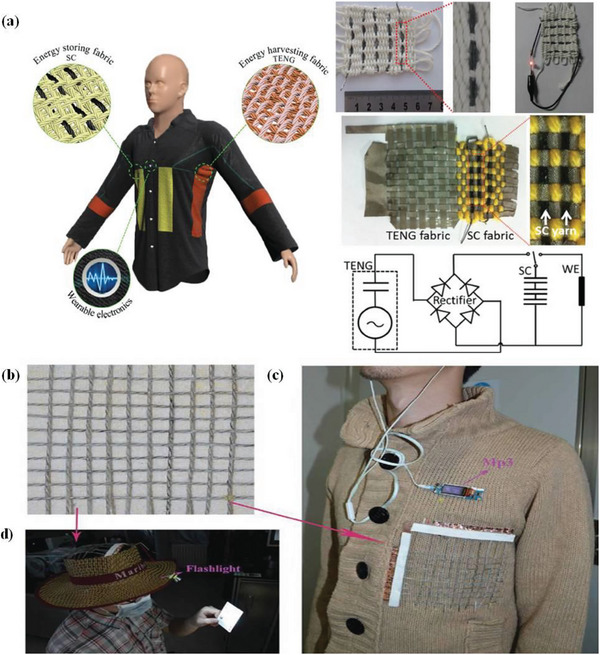
a) A self‐charging power textile that integrates supercapacitor yarns as energy‐storing fabrics, triboelectric nanogenerator (TENG) cloth as energy‐harvesting fabrics, and wearable electronics. Reproduced with permission.^[^ ^126^ ^]^ Copyright 2016, Wiley‐VCH. b) The asymmetric wire supercapacitors can be weaved as a textile to be integrated with everyday clothing to power c) an MP3 player and d) an LED flashlight. Reproduced with permission.^[^ ^130^ ^]^ Copyright 2016, Wiley‐VCH.

Several conventional methods for fabricating fibers have disadvantages such as heavier weight of metal wires, higher cost of graphene fibers and poor conductivity of dip‐coated fabric yarns. Therefore, fabric yarn with a conformal thin metal coating is an ideal material for a 1D yarn supercapacitor because it maintains lightness and mechanical flexibility while also retaining high fabric conductivity.^[^ ^125^ ^]^ As an example, Pu et al.^[^ ^126^ ^]^ reported the manufacture of a simple and scalable all‐solid‐state flexible yarn supercapacitor, which can be integrated with a triboelectric nanogenerator (TENG) cloth for self‐charging power textiles (**Figure** **11**). In various energy‐harvesting devices, TENGs have been proven to collect mechanical energy from human body motion with high efficiency and high output PD.^[^ ^127^, ^128^ ^]^ In contrast to thermoelectric and photovoltaic energy generation, mechanical energy acquisition is virtually independent of weather and working conditions. In addition, due to their simple structure and huge material selection, mechanical energy harvesting devices are easy to design into woven fabric.^[^ ^129^ ^]^ To form the flexible yarn supercapacitors, a conformal Ni layer and RGO film were sequentially coated on the surface of a conventional polyester yarn (noted as RGO‐Ni‐yarn). The symmetrical yarn supercapacitor realized high capacitance (13.0 mF cm^−1^, 72.1 mF cm^−2^) and excellent cycle stability (96 cycles, capacitance retention of 96%). In addition, no significant reduction was observed after 1000 cycles of 180° bending. As a result, cost‐effective and industrially scalable methods for realizing yarn supercapacitors and self‐charging power textiles have paved the way for wearable electronic devices.

**Figure 12:**
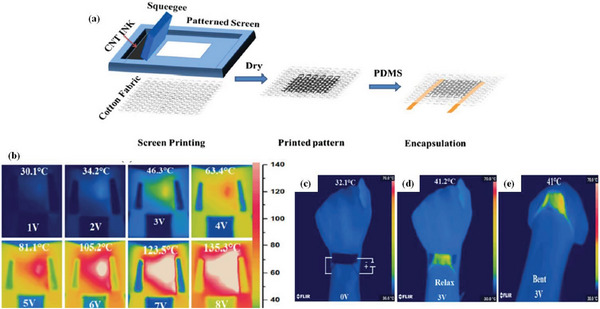
a) Schematic diagram of the preparation of CCCF. Heating performance of CCCF: b) infrared thermal images of CCCF under applied different voltages; c) CNT cotton composite fabric as a component of a wearable heater attached to the wrist; d–e) temperature profiles upon relaxing and bending of the wrist. Reproduced with permission.^[^ ^131^ ^]^ Copyright 2019, Elsevier.

As another example, Ai et al.^[^ ^130^ ^]^ synthesized a kind of CoNiO_2_ nanowire@carbon fiber composite electrode that showed a high specific capacitance of 794 F g^−1^ at a current density of 2 A g^−1^ and displayed a long cycle life by maintaining of 94% of the initial capacitance during 10000 cycles. They also used this composite electrode and an activated carbon electrode to prepare an asymmetric fiber‐type supercapacitor with a length of 1.2 meters. The device provided high specific capacitance of 1.68 mF cm^−1^ at 0.05 mA cm^−1^ and high ED of 0.95 mWh cm^−3^. At the same time, asymmetric wire‐supercapacitors (AWSs) have excellent mechanical properties, electrochemical stability, and energy decay does not occur during cycling under different bending conditions. AWSs can be used to generate power on straps, portable devices, textiles and belts. As shown in Figure 11b–d, AWSs can be integrated into clothing and used as a power source for portable electronic devices such as MP3 players and mobile phones. Linear supercapacitors can also be made into belts, replacing traditional belts or straps in some electronic devices such as electronic watches, thus playing two roles. While AWSs have a shorter power supply time than traditional batteries, AWSs have faster charging rates, higher PD and longer cycle life than batteries. At the same time, manufacturing linear supercapacitors is much simpler than linear cells and does not require complicated equipment. Therefore, linear supercapacitors are an attractive candidate for future wearable electronic devices. Further, Sadi et al.^[^ ^131^ ^]^ developed a multifunctional cotton composite fabric by incorporating an aqueous CNT dispersion into cotton fabric by screen printing (**Figure** **12**). The potential of composite fabrics for electrical heating performance as wearable heating devices was investigated. The CNT/Cotton composite fabric (CCCF) showed excellent heating properties (2 × 9 cm^2^) when a voltage (6 V) was applied to the fabric. As shown in Figure 12b, as the applied voltage was increased (1–8 V), more heat was created, and the image of the CCCF from the thermal imaging camera changed from blue to red, indicating that the surface temperature of the CCCF was gradually rising. Figure 12c–e shows the CCCF as part of a wearable heating device. When no voltage was applied, a uniform temperature distribution (32.1 °C) was observed on the CCCF surface. When a voltage of 3 V was applied, the temperature elsewhere in the hand did not change, but the temperature of the wrist area rose to ≈41.2 °C, and only a slight change in temperature (41 °C) was observed when the wrist was bent, showing that the CCCF continued to operate during exercise and still maintained consistent heating performance. The superior heating performance of the CCCF demonstrates its potential in wearable heating devices.

#### 2.3.3 FSCs in Sensing Medium

**Figure 13:**
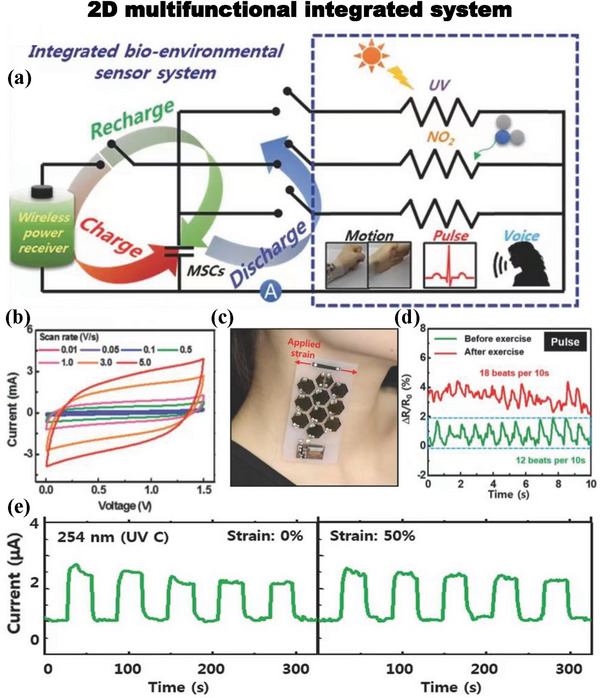
a) Circuit diagram of a 2D multifunctional integrated system consisting of radio‐frequency power receiver, MSC array, strain sensor, and UV/NO_2_ gas sensor. b) CV curves of the MSC. c) Photograph of the multifunctional integrated system. d) Carotid pulse curve. e) Current response of the multifunctional integrated system under stretching while exposed to 254 nm light. Reproduced with permission.^[^ ^147^ ^]^ Copyright 2016, Wiley‐VCH.

The combination of FSCs and sensing devices also makes our lives increasingly convenient and smarter.^[^ ^72^ ^]^ Bi et al. developed a planar‐type flexible integrated photodetector system driven by a flake MSC. In their work, the fabricated MSC exhibited a volumetric capacitance of 8.01 F cm^−3^ and an ED of 6.204 Wh cm^−3^. Further, Ha et al.^[^ ^147^ ^]^ reported a connectable and scalable multi‐sensor that can be integrated with an MSC array. **Figure** **13** shows a circuit diagram of a 2D multifunctional integrated system, including an MSC array, a strain sensor, a UV/NO_2_ gas sensor and a radio‐frequency power receiver. The CV curves of the MSC are shown in Figure 13b. The results show that the MSC has superior electrochemical performance with a volumetric capacitance of 4.7 F cm^−3^ and an ED of 1.5 mWh cm^−3^ at a PD of 12.6 Wh cm^−3^. Figure 13c shows a photograph of the integrated system attached directly to the tester's neck, indicating its stretchability and ability to be adhered to the body. Figure 13d shows the carotid pulse curve measured by a strained graphene foam (FGF) strain sensor. The FGF strain sensor has also been utilized to successfully detect swallowing, sounds and repeated movements of the body, indicative of great potential for detecting biological environmental signals.

#### 2.3.4 FSCs in Self‐powered Devices

**Figure 14:**
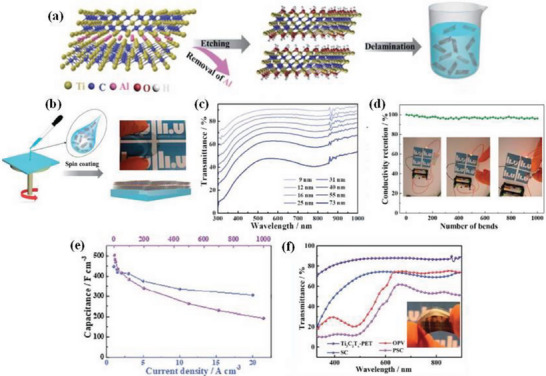
a) MXene prepared from Ti_3_AlC_2_ precursor using the MILD method. b) Schematic diagram of the preparation of transparent flexible electrodes. c) Transmission spectra of Ti_3_C_2_T_x_ films of various thicknesses. d) The Ti_3_C_2_T_x_ electrode on the PET substrate maintains conductivity when bent. The images show the conductivity of the Ti_3_C_2_T_x_ electrode is retained in the bent and twisted states. e) Volume capacitance obtained from GCD and CV. f) Transmittance of translucent PSCs with Ti_3_C_2_T_x_ as the transparent common electrode. Inset: a photograph of a transparent, flexible OPV device based on Ti_3_C_2_T_x_ electrodes. Reproduced with permission.^[^ ^145^ ^]^ Copyright 2020, Royal Society of Chemistry.

The rapid development of wearable electronic devices is improving people's daily lives through applications such as medical care,^[^ ^132^ ^]^ protection,^[^ ^133^, ^134^, ^135^ ^]^ health monitoring,^[^ ^136^, ^137^ ^]^ flexible energy supply systems,^[^ ^138^, ^139^ ^]^ and artificial intelligence.^[^ ^140^ ^]^ Stable self‐powered devices are the main challenge for wearable electronic devices. Energy storage devices, as an indispensable part of self‐powered devices, can effectively solve the problem of changes in light intensity on solar cells caused by the influences of day and night, weather and the seasons that otherwise result in unsustainable and unstable power supply to the equipment. Considering the requirements of safety and flexibility of wearable energy storage devices, conventional energy storage devices are not suitable due to their heaviness, large size, and rigidity, and it is difficult for them to meet the requirements of fast charging for wearable electronic devices. As promising energy‐storage devices, FSCs have attracted widespread attention in the field of wearable electronics due to their fast charging/discharging capabilities and long cycle life. Moreover, the emerging all‐solid‐state supercapacitors can be used as wearable electronic devices to easily meet the need for flexibility the ability to operate under various deformations (such as bending and twisting).^[^ ^123^, ^141^ ^]^ Meanwhile, the energy‐storage performance of wearable devices is also crucial. Nowadays, because of the advantage of their excellent flexibility, integrated systems containing supercapacitors have attracted widespread attention.^[^ ^141^, ^142^, ^143^ ^]^ As a typical example, Qin et al.^[^ ^145^ ^]^ obtained a flexible, transparent film by integrating Ti_3_C_2_T_x_ MXene in the vertical direction, using it as a common electrode to integrate organic photovoltaics (OPV) and transparent MXene supercapacitors, which is essential for the realization of flexible and printable electronic products in the future. The minimally intensive layer delamination (MILD) method shown in **Figure** **14**a was used to prepare MXene from the precursor Ti_3_AlC_2_, and thus a flexible transparent MXene film was prepared by spin coating, as shown in Figure 14b. This translucent film displayed excellent flexibility, transmittance and conductivity (Figure 14c,d), which led to a high PCE of 13.6% when used as the transparent electrode of an OPV device. As an active material for supercapacitors, it has a high volumetric specific capacity of 502 F cm^−3^ (Figure 14e). Finally, the translucent photovoltaic supercapacitors (PSCs) with Ti_3_C_2_T_x_ as the transparent common electrode had an AVT (average visible transmittance) of 33.5% and a maximum energy storage value of 88%, as shown in Figure 14f. This technology has a wide range of applications and can efficiently reduce the production cost of flexible PSCs.

**Figure 15:**
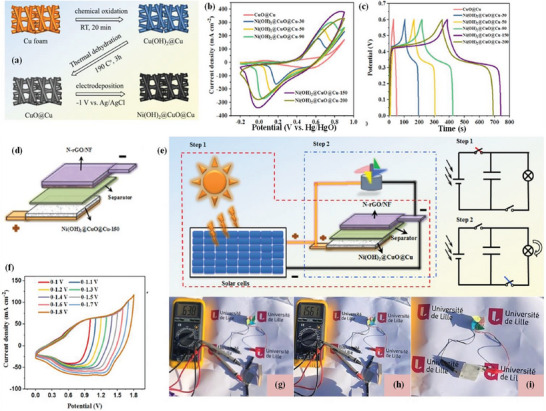
a) Synthesis diagram of a Ni(OH)_2_@CuO@Cu electrode. The electrochemical behavior of different electrodes was measured in 2 M KOH aqueous solution. b) CV curve collected at 20 mV s^−1^. (c) GCD profile of different electrodes collected at 20 mA cm^−2^. d) Schematic diagram of a compact hybrid supercapacitor device. e) Schematic diagram and equivalent circuit of the integrated system, which integrates solar cells and hybrid supercapacitor devices to operate a home‐made fan. f) CV plots measured at 100 mV^−1^ for different potential windows. g,h) Photographs of solar battery charging of hybrid supercapacitor equipment. i) Photos of installation and operation of a home‐made fan. Reproduced with permission.^[^ ^150^ ^]^ Copyright 2020, Elsevier.

In recent years, the research of solar cells has also developed rapidly,^[^ ^146^, ^147^, ^148^, ^149^ ^]^ so Li and co‐workers combined solar cells and hybrid supercapacitors to form an integrated system to drive the rotation of small fans, thereby realizing energy conversion, storage and utilization.^[^ ^150^ ^]^ The Ni(OH)_2_@CuO@Cu composite materials were fabricated by depositing Ni(OH)_2_ on CuO@Cu for different times by electrochemical deposition (**Figure** **15**a). The electrochemical performance of the prepared composite electrodes in 2 M KOH solution are shown in Figure 15b,c, and in all the prepared electrodes, Ni(OH)_2_ @ CuO@Cu‐150 at a current density of 20 mA cm^−2^ showed high areal capacity of 7063.2 mC cm^−2^. Therefore, using the Ni(OH)_2_@CuO@Cu‐150 composite electrode as an energy‐storage device assembled with a PD of 1.6 mW cm^−2^, a high areal ED of 130.4 µWh cm^−2^ was obtained. Figure 15f show the CV curves obtained from the device under different potential window tests at 100 mV s^−1^. Finally, the prepared hybrid supercapacitors and solar cells were combined into a built‐in integrated system for operating a home‐made fan (Figure 15e). After being charged by the solar battery, the hybrid supercapacitor device stored energy and further supplied power for the fan for 59 s, thus realizing energy conversion, storage and utilization. Therefore, it was demonstrated that the application of supercapacitors in self‐powered equipment has broad prospects.

## 3 Conclusions and Prospects

With the in‐depth research and rapid development of flexible wearable electronic devices, FSCs have received ever‐increasing attention. Considering the selection of electrode materials and substrates for FSCs, this review summarized several typical preparation methods and structural properties as well as the application progress of FSC electrode materials, which has lead to the following conclusions: 1) Carbon‐based materials are the better electrode candidates because of their high flexibility, which is the key research topic of FSC electrode materials. Although carbon‐based materials have better conductivity and rate performance, the lower theoretical specific capacitance of carbon‐based materials limits their development. Therefore, modification and compounding of carbon‐based materials should be the focus of future research. 2) Miniaturization of flexible electronic products will be a main research direction in the future and will involve making the electrodes smaller without losing ED; whereas designing nano‐scale electrode materials in order to provide more electrochemical reactive sites is the important direction for flexible electrodes. 3) With the development of electronic products, one‐dimensional fibers and two‐dimensional planar MSCs will be the main directions of future development. Simultaneously with the further miniaturization of devices, the development of gel electrolytes that perfectly match the electrode materials is particularly important. 4) Improving the packaging process of the devices is also an important direction for future research of flexible wearable supercapacitors.

At present, there are some unresolved problems in flexible wearable supercapacitors. These problems are opportunities even if the researchers are challenged: 1) 1D supercapacitors often use polymer gel electrolytes due to their structural particularity. Their low ionic conductivity and narrow potential window limit the energy storage and ED of the corresponding supercapacitors, so it is especially important to improve existing electrolytes and develop new gel electrolytes. 2) In most research reports of one‐dimensional supercapacitors, the length of the device is very limited. As the length of the device increases, the equivalent series resistance will increase accordingly, which will greatly reduce its electrochemical performance and cause the device to lose energy storage performance. However, the length of the device is very important in practical applications and textile processes, so new preparation methods to extend the length of the fiber electrode must be explored. 3) For flexible wearable supercapacitors, the match of mechanical properties, flexibility and electrochemical performance is crucial. The mechanical properties affect the durability of the device. The flexibility also determines comfort during use. Only when these three properties are perfectly matched will flexible wearable supercapacitors have more practical and far‐reaching potential. 4) The safety of flexible wearable supercapacitors has been of wide concern to the researchers as the electrolyte commonly used in supercapacitors often has a certain toxicity and corrosiveness. In addition, some nano‐electrode materials also have harmful effects on the human body. Supercapacitors should avoid negative impact on the human body. 5) There has not been a breakthrough in cleanable, flexible, wearable supercapacitors because the commonly used electrolytes are all soluble in water, so cleanable devices have not appeared. Therefore, it is an important challenge for researchers to study novel capacitor packaging technology, so that the resulting wearable devices can be washed like ordinary clothing while maintaining their normal electrochemical performance.

Although the above challenges exist in the field of flexible wearable supercapacitors, in recent years, with the increasing development of technology, researchers have also developed washable energy storage fabrics that are not only soft and breathable, but can also withstand repeated machine washing. While taking into account the performance, the research direction is also more inclined to be safe, harmless and pollution‐free. In addition, there are also reports of continuous preparation of different lengths of energy storage fibers, and they found that the internal resistance of energy storage fibers decreases with increasing length and eventually plateaus. This method of continuous preparation can effectively reduce the equivalent series resistance of one‐dimensional supercapacitors.

All in all, although supercapacitors currently face many challenges, we believe that supercapacitors have a bright future and will be ubiquitous in the future, especially in portable devices and wearable devices. In order to achieve final practical applications, researchers need to conduct more in‐depth research and screen out energy storage materials with good energy‐storage performance, safety and low cost. Perhaps one day, electronic communication devices and office equipment such as mobile phones, tablets, and laptops will stick to our bodies like skin, instead of being carried in heavy backpacks. Our glasses, watches, and rings will become small smart terminals, which will make our lives more convenient. Therefore, we hope that more researchers can devote themselves to exploring the field of supercapacitors that can change our lives in the future.

## Conflict of Interest

The authors declare no conflict of interest.

## References

[1] M. Yu , T. Zhai , X. Lu , X. Chen , S. Xie , W. Li , C. Liang , W. Zhao , L. Zhang , Y. Tong , J. Power Sources 2013, 239, 64.

[2] J. H. Lee , G. Yang , C. H. Kim , R. L. Mahajan , S. Y. Lee , S. J. Park , Energy Environ. Sci. 2022, 15, 2233.

[3] Q. Zhou , X. Chen , F. Su , X. Lyu , M. Miao , Ind. Eng. Chem. Res. 2020, 59, 5752.

[4] R. Reece , C. Lekakou , P. A. Smith , ACS Appl. Mater. Interfaces 2020, 12, 25683. 32407618 10.1021/acsami.9b23427

[5] H. Y. Jung , Y. R. Kim , H. T. Jeong , Carbon Lett. 2020, 30, 107.

[6] Q. Xue , J. Sun , Y. Huang , M. Zhu , Z. Pei , H. Li , Y. Wang , N. Li , H. Zhang , C. Zhi , Small 2017, 13, 1701827. 10.1002/smll.201701827 28941073

[7] P. Li , Z. Jin , L. Peng , F. Zhao , D. Xiao , Y. Jin , G. Yu , Adv. Mater. 2018, 30, 1800124. 10.1002/adma.201800124 29582483

[8] Q. Wang , Y. Ma , X. Liang , D. Zhang , M. Miao , Chem. Eng. J. 2019, 371, 145.

[9] K. Zhang , H. Hu , W. Yao , C. Ye , J. Mater. Chem. A 2015, 3, 617.

[10] S. L. Chou , J. Z. Wang , S. Y. Chew , H. K. Liu , S. X. Dou , Electrochem. Commun. 2008, 10, 1724.

[11] J. H. Sung , S. J. Kim , S. H. Jeong , E. H. Kim , K. H. Lee , J. Power Sources 2006, 162, 1467.

[12] V. L. Pushparaj , M. M. Shaijumon , A. Kumar , S. Murugesan , L. Ci , R. Vajtai , R. J. Linhardt , O. Nalamasu , P. M. Ajayan , Proc Natl Acad Sci U S A 2007, 104, 13574. 17699622 10.1073/pnas.0706508104 PMC1959422

[13] W. Gao , N. Singh , L. Song , Z. Liu , A. L. Reddy , L. Ci , R. Vajtai , Q. Zhang , B. Wei , P. M. Ajayan , Nat. Nanotechnol. 2011, 6, 496. 21804554 10.1038/nnano.2011.110

[14] Z. Yang , J. Deng , X. Chen , J. Ren , H. Peng , Angew Chem Int Ed Engl 2013, 52, 13453. 24214659 10.1002/anie.201307619

[15] J. Yu , W. Lu , S. Pei , K. Gong , L. Wang , L. Meng , Y. Huang , J. P. Smith , K. S. Booksh , Q. Li , J. H. Byun , Y. Oh , Y. Yan , T. W. Chou , ACS Nano 2016, 10, 5204. 27096412 10.1021/acsnano.6b00752

[16] D. Zhao , C. Chen , Q. Zhang , W. Chen , S. Liu , Q. Wang , Y. Liu , J. Li , H. Yu , Adv. Energy Mater. 2017, 7, 1700739.

[17] S. Zheng , X. Tang , Z. S. Wu , Y. Z. Tan , S. Wang , C. Sun , H. M. Cheng , X. Bao , ACS Nano 2017, 11, 2171. 28157332 10.1021/acsnano.6b08435

[18] D. W. Kim , S. M. Jung , H. Y. Jung , J. Mater. Chem. A 2020, 8, 532.

[19] J. Zhou , S. Zhang , Y. N. Zhou , W. Tang , J. Yang , C. Peng , Z. Guo , Electrochem. Energy Rev. 2021, 4, 219.

[20] A. H. Khadem , T. U. Hasan , A. N. M. M. Rahman , S. A. Smriti , S. Alimuzzaman , J. Energy Storage 2022, 56, 105988.

[21] X. Wang , Y. Ding , F. Chen , H. Lu , N. Zhang , M. Ma , ACS Appl. Energy Mater. 2018, 1, 5024.

[22] Z. Li , T. Huang , W. Gao , Z. Xu , D. Chang , C. Zhang , C. Gao , *ACS Nano*, 2017, 11, 11056–11065. 10.1021/acsnano.7b05092 29035519

[23] L. Wen , F. Li , H. M. Cheng , Adv. Mater. 2016, 28, 4306. 26748581 10.1002/adma.201504225

[24] Z. Li , Y. Mi , X. Liu , S. Liu , S. Yang , J. Wang , J. Mater. Chem. 2011, 21, 14706.

[25] J. Zhang , X. Yang , Y. He , Y. Bai , L. Kang , H. Xu , F. Shi , Z. Lei , Z. H. Liu , J. Mater. Chem. A 2016, 4, 9088.

[26] X. Chen , H. Lin , J. Deng , Y. Zhang , X. Sun , P. Chen , X. Fang , Z. Zhang , G. Guan , H. Peng , Adv. Mater. 2014, 26, 8126. 25338545 10.1002/adma.201403243

[27] L. Dong , C. Xu , Q. Yang , J. Fang , Y. Li , F. Kang , J. Mater. Chem. A 2015, 3, 4729.

[28] E. Wilson , M. F. Islam , ACS Appl. Mater. Interfaces 2015, 7, 5612. 25699583 10.1021/acsami.5b01384

[29] Y. P. Zhu , N. Li , T. Lv , Y. Yao , H. N. Peng , J. Shi , S. Cao , T. Chen , J. Mater. Chem. A 2018, 6, 941.

[30] Z. Yu , L. Chao , D. Abbitt , J. Thomas , J. Mater. Chem. A 2014, 2, 10923.

[31] J. Yin , K. Wei , J. Zhang , S. Liu , X. Wang , X. Wang , Q. Zhang , Z. Qin , T. Jiao , Cell Rep Phys Sci 2022, 3, 100893.

[32] K. Xiao , L. X. Ding , G. Liu , H. Chen , S. Wang , H. Wang , Adv. Mater. 2016, 28, 5997. 27158775 10.1002/adma.201601125

[33] D. Jiang , C. Li , W. Yang , J. Zhang , J. Liu , J. Mater. Chem. A 2017, 5, 18684.

[34] Z. Niu , W. Zhou , X. Chen , J. Chen , S. Xie , Adv. Mater. 2015, 27, 6002. 26316309 10.1002/adma.201502263

[35] P. Li , Y. Yang , E. Shi , Q. Shen , Y. Shang , S. Wu , J. Wei , K. Wang , H. Zhu , Q. Yuan , A. Cao , D. Wu , ACS Appl. Mater. Interfaces 2014, 6, 5228. 24621200 10.1021/am500579c

[36] J. L. Bideau , L. Viau , A. Vioux , Chem. Soc. Rev. 2011, 40, 907. 21180731 10.1039/c0cs00059k

[37] Y. Gu , S. Zhang , L. Martinetti , K. H. Lee , L. D. Mcintosh , C. D. Frisbie , T. P. Lodge , J. Am. Chem. Soc. 2013, 135, 9652. 23777188 10.1021/ja4051394

[38] X. H. Liu , D. B. Wu , H. L. Wang , Q. G. Wang , Adv. Mater. 2014, 26, 4370. 24737280 10.1002/adma.201400240

[39] L. Han , H. Huang , X. Fu , J. Li , Z. Yang , X. Liu , L. Pan , M. Xu , Chem. Eng. J. 2020, 392, 123733.

[40] M. Wang , L. Fan , G. Qin , X. Hu , Y. Wang , C. Wang , J. Yang , Q. Chen , J. Membr. Sci. 2020, 597, 117740.

[41] C. Choi , J. H. Kim , H. J. Sim , J. Di , R. H. Baughman , S. J. Kim , Adv. Energy Mater. 2017, 7, 1602021.

[42] Y. Meng , Y. Zhao , C. Hu , H. Cheng , Y. Hu , Z. Zhang , G. Shi , L. Qu , Adv. Mater. 2013, 25, 2326. 23463634 10.1002/adma.201300132

[43] P. Xu , B. Wei , Z. Cao , J. Zheng , K. Gong , F. Li , J. Yu , Q. Li , W. Lu , J.‐H. Byun , ACS Nano 2015, 9, 6088. 25961131 10.1021/acsnano.5b01244

[44] Z. Zhang , J. Deng , X. Li , Z. Yang , H. Peng , Adv. Mater. 2015, 27, 356. 25424189 10.1002/adma.201404573

[45] H. T. Jeong , Carbon Lett. 2020, 30, 55.

[46] A. M. Gaikwad , A. M. Zamarayeva , J. Rousseau , H. Chu , I. Derin , D. A. Steingart , Adv. Mater. 2012, 24, 5071. 22760812 10.1002/adma.201201329

[47] M. Shin , J. H. Song , G. H. Lim , B. Lim , J. J. Park , U. Jeong , Adv. Mater. 2014, 26, 3706. 24664816 10.1002/adma.201400009

[48] Z. Yang , J. Deng , X. Chen , J. Ren , H. Peng , Angew. Chem., Int. Ed. 2013, 52, 13453. 10.1002/anie.201307619 24214659

[49] B. Zhu , E. W. C. Chan , S. Y. Li , X. Sun , J. Travas‐Sejdic , J. Mater. Chem. C 2022, 10, 14882.

[50] J. Huang , S. Han , J. Zhu , Q. Wu , H. Chen , A. Chen , J. Zhang , B. Huang , X. Yang , L. Guan , Adv. Func. Mater. 2022, 32, 2205708.

[51] J. Y. Sun , C. Keplinger , G. M. Whitesides , Z. Suo , Adv. Mater. 2014, 26, 7608. 25355528 10.1002/adma.201403441

[52] M. Hu , J. Wang , J. Liu , J. Zhang , X. Ma , Y. Huang , Chem. Commun. 2018, 54, 6200. 10.1039/c8cc03375g 29850729

[53] X. Liang , K. Nie , X. Ding , L. Dang , J. Sun , F. Shi , H. Xu , R. Jiang , X. He , Z. Liu , Z. Lei , ACS Appl. Mater. Interfaces 2018, 10, 10087. 29508993 10.1021/acsami.7b19043

[54] X. Liu , D. Wu , H. Wang , Q. Wang , Adv. Mater. 2014, 26, 4370. 24737280 10.1002/adma.201400240

[55] Y. Huang , M. Zhong , Y. Huang , M. Zhu , Z. Pei , Z. Wang , Q. Xue , X. Xie , C. Zhi , Nat. Commun. 2015, 6, 10310. 26691661 10.1038/ncomms10310 PMC4703889

[56] Y. Huang , M. Zhong , F. K. Shi , X. Y. Liu , Z. Tang , Y. Wang , Y. Huang , H. Q. Hou , X. Xie , C. Zhi , Angew.Chem. Int. Ed. 2017, 56, 9141. 10.1002/anie.201705212 28631401

[57] X. Liu , N. Wen , X. Wang , Y. Zheng , ACS Sustainable Chem. Eng. 2015, 3, 475.

[58] Q. Qu , S. Yang , X. Feng , Adv. Mater. 2011, 23, 5574. 22052661 10.1002/adma.201103042

[59] Z. Shi , L. Xing , Y. Liu , Y. Gao , J. Liu , Carbon 2018, 129, 819.

[60] P. Hao , J. Tian , Y. Sang , C. C. Tuan , G. Cui , X. Shi , C. P. Wong , B. Tang , H. Liu , Nanoscale 2016, 8, 16292. 27714086 10.1039/c6nr05385h

[61] C. Yan , M. Jin , X. Pan , L. Ma , X. Ma , RSC Adv. 2020, 10, 9299. 35497250 10.1039/c9ra10701k PMC9050157

[62] C. Yu , C. Masarapu , J. Rong , B. Wei , H. Jiang , Adv. Mater. 2010, 21, 4793. 10.1002/adma.200901775 21049496

[63] D. Qi , Z. Liu , Y. Liu , R. L. Wan , B. Zhu , H. Yang , J. Yu , W. Wang , H. Wang , S. Yin , Adv. Mater. 2015, 27, 5559. 26291187 10.1002/adma.201502549

[64] Y. Zhou , X. Hu , Y. Y. Shang , C. Hua , P. Song , X. Li , Y. Zhang , A. Cao , RSC Adv. 2016, 6, 62062.

[65] J. Chen , D. Shi , Z. Yang , W. Dong , M. Chen , J. Power Sources 2022, 532, 231326.

[66] D. P. Dubal , N. R. Chodankar , Z. Caban‐Huertas , F. Wolfart , M. Vidotti , R. Holze , C. D. Lokhande , P. Gomez‐Romero , J. Power Sources 2016, 308, 158.

[67] H. Cheng , Z. Dong , C. Hu , Y. Zhao , Y. Hu , L. Qu , N. Chen , L. Dai , Nanoscale 2013, 5, 3428. 23475309 10.1039/c3nr00320e

[68] F. Jiang , Y. Fang , Q. Xue , L. Chen , Y. Lu , Mater. Lett. 2010, 64, 199.

[69] X. B. Chen , J. G. Ding , J. Jiang , G. Zhuang , Z. H. Zhang , P. Z. Yang , RSC Adv. 2018, 8, 29488. 35547327 10.1039/c8ra05158e PMC9085153

[70] H. Li , J. He , X. Cao , L. Kang , X. He , H. Xu , F. Shi , R. Jiang , Z. Lei , Z. H. Liu , J. Power Sources 2017, 371, 18.

[71] S. Ullah , J. Yu , H. Liu , W. Iqbal , B. Yang , C. Li , C. Zhu , J. Xu , Appl. Surf. Sci. 2019, 487, 180.

[72] D. He , L. Wu , Y. Yao , J. Zhang , Z. H. Huang , M. X. Wang , Appl. Surf. Sci. 2020, 507, 145108.

[73] E. Frackowiak , V. Khomenko , K. Jurewicz , K. Lota , F. Béguin , J. Power Sources 2006, 153, 413.

[74] K. Wang , H. Wu , Y. Meng , Z. Wei , Small 2014, 10, 14. 23959804 10.1002/smll.201301991

[75] H. B. Zhao , L. Yuan , Z. B. Fu , C. Y. Wang , X. Yang , J. Y. Zhu , J. Qu , H. B. Chen , D. A. Schiraldi , ACS Appl. Mater. Interfaces 2016, 8, 9917. 27045343 10.1021/acsami.6b00510

[76] N. Kurra , B. Ahmed , Y. Gogotsi , H. N. Alshareef , Adv. Energy Mater. 2016, 6, 1601372.

[77] M. A. Bissett , S. D. Worrall , I. A. Kinloch , R. A. W. Dryfe , Electrochim. Acta 2016, 201, 30.

[78] M. Wu , L. Zhang , D. Wang , C. Xiao , S. Zhang , J. Power Sources 2008, 175, 669.

[79] C. Huang , N. P. Young , P. S. Grant , J. Mater. Chem. A 2014, 2, 11022.

[80] L. Jiang , X. Lu , J. Xu , Y. Chen , G. Wan , Y. Ding , J Mater Sci 2015, 26, 747.

[81] L. Gao , J. Song , J. U. Surjadi , K. Cao , Y. Han , D. Sun , X. Tao , Y. Lu , ACS Appl. Mater. Interfaces 2018, 10, 28597. 30036032 10.1021/acsami.8b08680

[82] G. Sun , X. Zhang , R. Lin , J. Yang , H. Zhang , P. Chen , Angew. Chem., Int. Ed. 2015, 54, 4651. 10.1002/anie.201411533 25694387

[83] J. T. Carvalho , I. Cunha , J. Coelho , E. Fortunato , R. Martins , L. Pereira , ACS Appl. Energy Mater. 2022, 5, 11987. 36311466 10.1021/acsaem.2c01222 PMC9597547

[84] S. Seyedin , E. R. S. Yanza , J. M. Razal , J. Mater. Chem. A 2017, 5, 24076.

[85] Z. Wang , S. Qin , S. Seyedin , J. Zhang , J. Wang , A. Levitt , N. Li , C. Haines , R. Ovalle‐Robles , W. Lei , Y. Gogotsi , R. H. Baughman , J. M. Razal , Small 2018, 14, 5467. 10.1002/smll.201802225 30084530

[86] C. Gao , Z. Gao , Y. Wei , N. Luo , Y. Liu , P. Huo , ACS Appl. Mater. Interfaces 2023, 15, 2951. 36597008 10.1021/acsami.2c18935

[87] Q. Li , M. Liu , F. Huang , X. Zuo , X. Wei , S. Li , H. Zhang , Chem. Eng. J. 2022, 437, 135494.

[88] Y. Wang , N. Chen , Y. Liu , X. Zhou , B. Pu , Y. Qing , M. Zhang , X. Jiang , J. Huang , Q. Tang , B. Zhou , W. Yang , Chem. Eng. J. 2022, 450, 138398.

[89] H. Li , S. Lin , H. Li , Z. Wu , Q. Chen , L. Zhu , C. Li , X. Zhu , Y. Sun , Small Methods 2022, 6, 2101320. 10.1002/smtd.202101320 35032157

[90] X. Li , J. Wang , Y. Zhao , F. Ge , S. Komarneni , Z. Cai , ACS Appl. Mater. Interfaces 2016, 8, 25905. 27618744 10.1021/acsami.6b06156

[91] L. Dong , C. Xu , Y. Li , Z. Pan , G. Liang , E. Zhou , F. Kang , Q. H. Yang , Adv. Mater. 2016, 28, 9313. 27572268 10.1002/adma.201602541

[92] K. Wang , B. Zheng , M. Mackinder , N. Baule , H. Qiao , H. Jin , T. Schuelke , Q. H. Fan , Energy Storage Mater. 2019, 20, 299.

[93] Z. Ren , Y. Li , J. Yu , iScience 2018, 9, 138. 30391849 10.1016/j.isci.2018.10.016 PMC6215974

[94] X. Wang , B. Liu , R. Liu , Q. Wang , X. Hou , D. Chen , R. Wang , G. Shen , Angew.Chem. Int. Ed. 2014, 53, 1849. 10.1002/anie.201307581 24505005

[95] Y. Chen , B. Xu , J. Wen , J. Gong , T. Hua , C. W. Kan , J. Deng , Small 2018, 14, 1704373. 10.1002/smll.201704373 29675877

[96] D. J. Ahirrao , H. M. Wilson , N. Jha , Appl. Surf. Sci. 2019, 491, 765.

[97] R. Zhong , M. Xu , N. Fu , R. Liu , A. Zhou , X. Wang , Z. Yang , Electrochim. Acta 2020, 348, 136209.

[98] B. Naresh , T. N. V. Krishna , S. Rao , H. J. Kim , Mater. Lett. 2019, 248, 218.

[99] M. S. Kim , J. W. Kim , J. Yun , Y. R. Jeong , S. W. Jin , G. Lee , H. Lee , D. S. Kim , K. Keum , J. S. Ha , Appl. Surf. Sci. 2020, 515, 146018.

[100] C. Wu , T. Zhou , Y. Du , S. Dou , H. Zhang , L. Jiang , Q. Cheng , Nano Energy 2019, 58, 517.

[101] X. Li , Y. Tang , J. Song , W. Yang , M. Wang , C. Zhu , W. Zhao , J. Zheng , Y. Lin , Carbon 2018, 129, 236.

[102] S. Verma , V. Gupta , A. Khosla , S. Kumar , S. Arya , Nanotechnology 2020, 31, 245401. 32109899 10.1088/1361-6528/ab7b07

[103] J. P. Chen , W. Huang , Z. Y. Jiang , J. L. Xu , S. Q. Zhao , Y. H. Liu , J. Phys. D: Appl. Phys. 2020, 53, 165501.

[104] C. Xiong , M. Li , W. Zhao , C. Duan , Y. Ni , Journal of Materiomics 2020, 6, 523.

[105] D. Huang , Z. Lu , X. Liu , J. Gao , Z. Chen , X. Wang , X. Fu , Appl. Surf. Sci. 2022, 605, 154707.

[106] X. Zang , Q. Chen , P. Li , Y. He , X. Li , M. Zhu , X. Li , K. Wang , M. Zhong , D. Wu , H. Zhu , Small 2014, 10, 2583. 24648143 10.1002/smll.201303738

[107] L. Chen , L. Chen , Q. Ai , D. Li , P. Si , J. Feng , L. Zhang , Y. Li , J. Lou , L. Ci , Chem. Eng. J. 2018, 334, 184.

[108] S. Cho , J. Lim , Y. Seo , ACS Omega 2022, 7, 37825. 36312342 10.1021/acsomega.2c04822 PMC9609059

[109] M. M. Ovhal , N. Kumar , S. Lim , J. W. Kang , Appl. Surf. Sci. 2020, 529, 147072.

[110] X. Shi , S. Pei , F. Zhou , W. Ren , H. M. Cheng , Z. S. Wu , X. Bao , Energy Environ. Sci. 2019, 12, 1534.

[111] R. Cao , X. Pu , X. Du , W. Yang , J. Wang , H. Guo , S. Zhao , Z. Yuan , C. Zhang , C. Li , Z. L. Wang , ACS Nano 2018, 12, 5190. 29771494 10.1021/acsnano.8b02477

[112] C. Ma , L. Wu , M. Dirican , H. Cheng , J. Li , Y. Song , J. Shi , X. Zhang , Appl. Surf. Sci. 2021, 537, 147914.

[113] A. S. Ghouri , R. Aslam , M. S. Siddiqui , S. K. Sami , Front. Mater. 2020, 7, 1.

[114] W. Luo , X. Li , J. Y. Chen , J Ind Text 2020, 49, 1061.

[115] Z. Xu , C. Gao , Acc. Chem. Res. 2014, 47, 1267. 24555686 10.1021/ar4002813

[116] N. Sheng , S. Chen , J. Yao , F. Guan , M. Zhang , B. Wang , Z. Wu , P. Ji , H. Wang , Chem. Eng. J. 2019, 368, 1022.

[117] W. Zeng , L. Shu , Q. Li , S. Chen , F. Wang , X. M. Tao , Adv. Mater. 2014, 26, 5310. 24943999 10.1002/adma.201400633

[118] Z. Lou , S. Chen , L. Wang , R. Shi , L. Li , K. Jiang , D. Chen , G. Shen , Nano Energy 2017, 38, 28.

[119] J. Feng , Z. Yang , D. Yang , X. Ren , X. Zhu , Z. Jin , W. Zi , Q. Wei , S. Liu , Nano Energy 2017, 36, 1.

[120] L. Li , C. Fu , Z. Lou , S. Chen , W. Han , K. Jiang , D. Chen , G. Shen , Nano Energy 2017, 41, 261.

[121] Y. Li , Z. Lu , B. Xin , Y. Liu , Y. Cui , Y. Hu , Appl. Surf. Sci. 2020, 528, 146975.

[122] F. Zhang , J. Chen , G. G. Wallace , J. Yang , Prog. Mater. Sci. 2023, 133, 101069.

[123] T. Qin , S. Peng , J. Hao , Y. Wen , Z. Wang , X. Wang , D. He , J. Zhang , J. Hou , G. Cao , Adv. Energy Mater. 2017, 7, 1700409.

[124] M. S. Lal , S. Ramaprabhu , J. Electrochem. Soc. 2022, 169, 020514.

[125] W. Xiong , K. Hu , Z. Li , Y. Jiang , Z. Li , Z. Li , X. Wang , Nano Energy 2019, 66, 104149.

[126] X. Pu , L. Li , M. Liu , C. Jiang , C. Du , Z. Zhao , W. Hu , Z. L. Wang , Adv. Mater. 2016, 28, 98. 26540288 10.1002/adma.201504403

[127] Z. L. Wang , Faraday Discuss. 2014, 176, 447. 25406406 10.1039/c4fd00159a

[128] Z. L. Wang , ACS Nano 2013, 7, 9533. 24079963 10.1021/nn404614z

[129] X. Pu , L. Li , H. Song , C. Du , Z. Zhao , C. Jiang , G. Cao , W. Hu , Z. L. Wang , Adv. Mater. 2015, 27, 2472. 25736078 10.1002/adma.201500311

[130] Y. Ai , Z. Lou , L. Li , S. Chen , H. S. Park , Z. M. Wang , G. Shen , Adv. Mater. Technol. 2016, 1, 1600142.

[131] M. S. Sadi , M. Yang , L. Luo , D. Cheng , G. Cai , X. Wang , Cellulose 2019, 26, 6179.

[132] J. Kim , A. S. Campbell , B. E. F. Ávila , J. Wang , Nat. Biotechnol. 2019, 37, 389. 30804534 10.1038/s41587-019-0045-y PMC8183422

[133] B. Ciui , A. Martin , R. K. Mishra , T. Nakagawa , T. J. Dawkins , M. Lyu , C. Cristea , R. Sandulescu , J. Wang , ACS Sens. 2018, 3, 2375. 30226368 10.1021/acssensors.8b00778

[134] Q. Zhang , Q. Liang , Z. Zhang , Z. Kang , Q. Liao , Y. Ding , M. Ma , F. Gao , X. Zhao , Y. Zhang , Adv. Funct. Mater. 2018, 28, 1703801.

[135] Y. Jiang , J. Ma , J. Lv , H. Ma , H. Xia , J. Wang , C. Yang , M. Xue , G. Li , N. Zhu , ACS Sens. 2019, 4, 152. 30584759 10.1021/acssensors.8b01111

[136] Y. Lu , K. Jiang , D. Chen , G. Shen , Nano Energy 2019, 58, 624.

[137] J. Ma , Y. Jiang , L. Shen , H. Ma , T. Sun , F. Lv , A. Kiran , N. Zhu , Biosens. Bioelectron. 2019, 144, 111637. 31494509 10.1016/j.bios.2019.111637

[138] P. Sun , M. Qiu , M. Li , W. Mai , G. Cui , Y. Tong , Nano Energy 2019, 55, 506.

[139] Y. Yang , Q. Huang , L. Niu , D. Wang , C. Yan , Y. She , Z. Zheng , Adv. Mater. 2017, 29, 1606679. 10.1002/adma.201606679 28234421

[140] K. H. Yu , A. L. Beam , I. S. Kohane , Nat. Biomed. Eng. 2018, 2, 719. 31015651 10.1038/s41551-018-0305-z

[141] Q. Liao , N. Li , S. Jin , G. Yang , C. Wang , ACS Nano 2015, 9, 5310. 25938705 10.1021/acsnano.5b00821

[142] Y. Yuan , Y. Lu , B. E. Jia , H. Tang , L. Chen , Y. J. Zeng , Y. Hou , Q. Zhang , Q. He , L. Jiao , J. Leng , Z. Ye , J. Lu , Nano‐Micro Lett. 2019, 11, 42. 10.1007/s40820-019-0274-0 PMC7770920 34137998

[143] Z. Liu , Y. Zhong , B. Sun , X. Liu , J. Han , T. Shi , Z. Tang , G. Liao , ACS Appl. Mater. Interfaces 2017, 9, 22361. 28614655 10.1021/acsami.7b01471

[144] Y. Yuan , Y. Wu , T. Zhang , H. Tang , L. Meng , Y. J. Zeng , Q. Zhang , Z. Ye , J. Lu , J. Power Sources 2018, 404, 118.

[145] L. Q. Qin , J. X. Jiang , Q. Z. Tao , C. F. Wang , I. Persson , M. Fahlman , O. A. Persson , L. Hou , J. Rosen , F. L. Zhang , J. Materi. Chem. A 2020, 8, 5467.

[146] B. Li , Y. Kawakita , Y. Liu , M. Wang , M. Matsuura , K. Shibata , S. Ohira‐Kawamura , T. Yamada , S. Lin , K. Nakajima , S. Liu , Nat. Commun. 2017, 8, 16086. 28665407 10.1038/ncomms16086 PMC5497077

[147] J. Yan , H. Wu , H. Chen , L. Pang , Y. Zhang , R. Jiang , L. Li , S. Liu , Appl Catal B 2016, 194, 74.

[148] K. Wang , Z. Li , F. Zhou , H. Wang , H. Bian , H. Zhang , Q. Wang , Z. Jin , L. Ding , S. Liu , Adv. Energy Mater. 2019, 9, 1902529.

[149] L. Li , T. Zhang , J. Yan , X. Cai , S. Liu , Small 2017, 13, 1700441. 10.1002/smll.201700441 28508567

[150] M. Li , A. Addad , P. Roussel , S. Szunerits , R. Boukherroub , J. Colloid Interface Sci. 2020, 579, 520. 32623118 10.1016/j.jcis.2020.06.092

[151] D. Kim , D. Kim , H. Lee , Y. R. Jeong , J. S. Ha , Adv. Mater. 2016, 28, 748. 26641239 10.1002/adma.201504335

[152] Y. F. Ai , Z. Lou , S. Chen , D. Chen , Z. M. Wang , K. Jiang , G. Z. Shen , Nano Energy 2017, 35, 121.

